# On the importance of evolving phenotype distributions on evolutionary diversification

**DOI:** 10.1371/journal.pcbi.1008733

**Published:** 2021-02-16

**Authors:** Gil Jorge Barros Henriques, Koichi Ito, Christoph Hauert, Michael Doebeli

**Affiliations:** 1 Department of Zoology, University of British Columbia, Vancouver, British Columbia, Canada; 2 Department of Mathematics, University of British Columbia, Vancouver, British Columbia, Canada; Eotvos Lorand Tudomanyegyetem Termeszettudomanyi Kar, HUNGARY

## Abstract

Evolutionary branching occurs when a population with a unimodal phenotype distribution diversifies into a multimodally distributed population consisting of two or more strains. Branching results from frequency-dependent selection, which is caused by interactions between individuals. For example, a population performing a social task may diversify into a cooperator strain and a defector strain. Branching can also occur in multi-dimensional phenotype spaces, such as when two tasks are performed simultaneously. In such cases, the strains may diverge in different directions: possible outcomes include division of labor (with each population performing one of the tasks) or the diversification into a strain that performs both tasks and another that performs neither. Here we show that the shape of the population’s phenotypic distribution plays a role in determining the direction of branching. Furthermore, we show that the shape of the distribution is, in turn, contingent on the direction of approach to the evolutionary branching point. This results in a distribution–selection feedback that is not captured in analytical models of evolutionary branching, which assume monomorphic populations. Finally, we show that this feedback can influence long-term evolutionary dynamics and promote the evolution of division of labor.

## Author summary

Evolutionary branching is the phenomenon by which frequency-dependent selection causes a population with a unimodal phenotype distribution to diversify into multiple modes (strains). Simultaneous evolutionary changes in several traits results in divergence in different phenotypic directions. Here we show that branching preferentially occurs perpendicular to the direction of approach to the branching point. Employing different numerical techniques we demonstrate that this is due to changes in the shape of the population’s phenotypic distribution as it approaches the branching point: the population accumulates variance orthogonally to the selection gradient, which results in a predictable branching direction. The feedback between selection and the phenotypic distribution drives the preferred direction of branching, and ultimately affects the evolutionary equilibrium. For example, when populations are engaged in simultaneous snowdrift games, some branching directions promote division of labor. Thus, keeping track of the distribution is crucial to determine future states of the population.

## 1 Introduction

Natural selection can act on multiple traits at the same time. When this is the case, the population’s response to selection depends not only on the strength and direction of selection but also on the shape of its phenotypic distribution [1]. But selection also changes the shape of this distribution, which in turn may affect future dynamics. This creates the potential for a distribution–selection feedback that could render the outcome of evolutionary processes contingent on initial conditions and more generally on past evolutionary dynamics. However, mathematical models often make simplifying assumptions, such as monomorphic populations, that erase the influence of the population distribution. In this article, we explore one such situation that arises in the context of evolutionary branching, the process by which adaptive evolution splits a population into multiple strains [2–4]. Most analytical models of evolutionary branching disregard the phenotypic distribution; however, we show that, in multidimensional phenotype spaces, the direction of branching can be influenced by the population’s phenotypic distribution, which in turn is affected by the direction of approach to the branching point.

Branching occurs when frequency-dependent selection, resulting from interactions between individuals of the same species (e.g., competition or cooperation) or of different species (e.g., predation or parasitism), leads to phenotypic divergence [5]. This is possible because frequency-dependent selection causes the fitness landscape experienced by a population to change as the population’s trait value evolves. Hence, it is possible for natural selection to drive a population toward a fitness minimum (called an evolutionary branching point). The population then splits into distinct phenotypic strains which diverge from one another.

When the phenotype space is unidimensional, the outcome of evolutionary branching is often entirely determined by the shape of the fitness landscape at the branching point, regardless of the state of the population when it arrives at this point (but see [6, 7], who have shown that population size can also affect the outcome of branching: small populations are unable to undergo branching due to demographic noise.) When the phenotype space is multidimensional, however, fitness-based criteria are often not sufficient to predict long-term dynamics. Genetic correlations between traits, imposed by genes acting pleiotropically or by linkage disequilibrium, affect the multidimensional stability conditions [8, 9]. Epistatic interactions between traits tend to destabilize equilibria, so that branching becomes more prevalent at higher dimensions [10, 11]. Finally, branching may occur *before* the population reaches the equilibrium, in directions that are orthogonal to the selection gradient, when disruptive selection overwhelms directional selection [11–14].

Here we show that the shape of the phenotypic distribution also plays a role in determining the direction of branching. The shape of this distribution can be characterized by the genetic variance-covariance matrix (the **G**-matrix), which plays a central role in quantitative genetics theory, since it influences a population’s ability to respond to selection [1]. However, the **G**-matrix is not necessarily stable in time, since the phenotypic distribution itself changes in response to selection [15] in ways that are not yet fully understood [16, 17]. A series of simulation studies (e.g., [18–22]) have revealed that the **G**-matrix may be stable under some regimes (including correlational and stabilizing selection, moving optima, low migration, and correlated mutational effects), but this is not always the case. Because the direction in phenotype space from which a population approaches the branching point can affect its phenotypic distribution, it can also affect the outcome of evolutionary branching. In particular, we show that branching tends to occur, *ceteris paribus*, along a direction that is perpendicular to the direction of approach to the branching point.

We investigate this phenomenon using, as a case study, the process of cooperative-based branching. Cooperative interactions are those in which individuals incur some cost to provide benefits that accrue equally to both interaction partners. They describe, for example, the production of common resources (public goods), such as invertase in yeast [23] or antibiotic resistance (indole) in *Escherichia coli* [24]. Pairwise cooperative interactions have been modeled as an evolving quantitative phenotypic trait by [25], in a model called continuous snowdrift game (csg). In this model, individuals pay a cost that increases with the amount of cooperative investment and receive a benefit that depends on the total amount of public good produced in their interaction pair. Depending on the shape of the cost and benefit functions, [25] showed that evolutionary branching may occur: the initially monomorphic population evolves to some intermediary investment level and subsequently splits into a high-investment ‘cooperator’ strain and a low-investment ‘defector’ strain.

While the csg provides one possible explanation for the evolutionary origin of cooperators and defectors, it focuses on the production of a single public good. However, real organisms are engaged simultaneously in several different interactions and tasks [26] each of which could be modeled as a public goods game [27, 28]. Because we are interested in branching in a multidimensional phenotype space, here we consider a simple extension of the csg for multiple dimensions: i.e., individuals play multiple continuous snowdrift games simultaneously, and their fitness is the sum of the payoffs from each game. In such a case, the branching point is a minimum of the fitness landscape in all directions, and hence the direction of branching is not predictable. Despite its unpredictability, the branching direction could have important biological consequences. For example, consider that the population is engaged in two simultaneous games and evolutionary branching occurs in both traits. One possible outcome is that the population may split into a strain that performs both tasks, and another that performs none. Alternatively, diversification may result in complementary strains, each of which produces only one of the public goods. This would result in division of labor, with individuals of different strains relying on one another to share the products in which they specialize. Other outcomes, such as more than two co-existing strains, are also possible.

In this manuscript, we show that the initial phenotype of the population (before it approaches the branching point) influences the direction of evolutionary branching. We argue that during its approach to the branching point, the population accumulates phenotypic variance in the direction that is orthogonal to movement (similar to the phenomenon described by [29]). When the population reaches the branching point, its phenotypic distribution, which is determined by past selection, in turn determines the direction of evolutionary branching. We also show that the direction of evolutionary branching has important long-term consequences, because it influences the stable state the population will eventually reach. In the csg case, this can determine whether the equilibrium population consists of two specialist strains or of a cooperator and a defector strain. Analytical methods that ignore the population’s phenotypic distribution are hence ill-prepared to predict the direction and outcome of evolutionary branching.

## 2 Methods

### 2.1 Model description

Our model is a simple extension of the one-dimensional continuous snowdrift game (csg) to multiple dimensions. Individuals live in a well-mixed population and their fitness is determined by the payoffs of pairwise interactions.

#### One-dimensional csg

In the original model [25], each individual is characterized by a phenotype 0 < *x* < *x*_max_, which determines its investment into a costly good that the individual produces during an interaction. This good is shared by both interacting partners, who receive the same amount of benefit, regardless of their contribution. Upon interacting with a partner with phenotype *x*, a focal individual with phenotype *x*′ receives a payoff *P*(*x*′, *x*) = *B*(*x*′ + *x*) − *C*(*x*′), where *C*(*x*) and *B*(*x*) determine, respectively, the cost of public good production and the benefit associated with its consumption. Both functions are assumed to be smooth and strictly increasing, satisfying *B*(0) = *C*(0) = 0.

#### Multi-dimensional csg

We expand the original game so that individuals are engaged in the production of more than one public good. Thus, instead of a single scalar phenotype, individuals are characterized by a *k*-dimensional vector *z* = 〈*z*_1_, ⋯, *z*_*k*_〉, whose elements quantify the investment into each of the *k* costly goods. Each good *i* ∈ [1, *k*] has its own cost, benefit, and payoff functions, *C*_*i*_(*z*_*i*_), *B*_*i*_(*z*_*i*_), and $P_{i}\left(z_{i}^{\prime },z_{i}\right)$. The payoffs associated with each public good are entirely independent, so that a focal individual with phenotype *z*′ interacting with a partner with phenotype *z* earns a payoff $P\left(z^{\prime },z\right)=\sum_{i=1}^{k}P\left(z_{i}^{\prime },z_{i}\right)$. As such, producing multiple goods incurs no trade-offs whatsoever; similarly, there is no synergism or discounting between each good’s benefits.

#### Simplifying assumptions

In most of the manuscript we will focus on the simplest possible version of this model, i.e., we consider only two public goods (with *z* = 〈*z*_1_, *z*_2_〉). Because we are interested in studying the effect of the initial phenotype on the branching process, we chose functions that ensure that the fitness landscape is always perfectly symmetric, thus removing any selective effects that may affect the branching direction. Therefore, we assume that the public goods have the same exact effect on fitness (*B*_1_(*x*) = *B*_2_(*x*) ≡ *B*(*x*) and *C*_1_(*x*) = *C*_2_(*x*) ≡ *C*(*x*)), so that they are entirely interchangeable. We relax both assumptions (*k* = 2 and equal fitness functions) later on. Following [25], we use quadratic cost and benefit functions, *C*_*i*_(*x*) = *c*_*i*_(*x* − *d*_*i*_*x*^2^) and *B*_*i*_(*x*) = *a*_*i*_(*x* − *b*_*i*_*x*^2^) (and we drop the index *i* ∈ {1, 2} whenever it causes no confusion). Cost and benefit must be strictly increasing, so we restrict these quadratic functions to values below their maxima. In particular, *z*_*i*_ must be smaller than the maximum of *B*(2*z*_*i*_) and *C*(*z*_*i*_), i.e., (*z*_*i*_)_max_ = min(1/(4*b*), 1/(2*d*)).

### 2.2 Adaptive dynamics

We use adaptive dynamics [2, 3, 30] to analyze the evolution of the investment strategies before branching and to determine conditions for branching. Given a monomorphic population, with resident phenotype *z*, the growth rate of a rare mutant strategy *z*′ is given by the invasion fitness [4], *w*(*z*′, *z*) = *P*(*z*′, *z*) − *P*(*z*, *z*). Then, the direction of evolution in phenotype space is given by the selection gradient, D(z), a vector whose *i*^th^ entry is given by $D_{i}(z)=\partial w(z^{\prime },z)/\partial z_{i}^{\prime }{|}_{z^{\prime }=z}$. The selection gradient points from the resident toward the steepest uphill direction of the fitness landscape, so that the time-dynamics of *z* is described by [30]

$$\begin{array}{c} \frac{dz}{dt}=m\left(z\right)A\left(z\right)D\left(z\right), \end{array}$$ (1)

where *m*(*z*) is the rate of occurrence of new mutations and $A\left(z\right)=(\begin{array}{rr} \sigma^{2} & 0\\ 0 & \sigma^{2} \end{array})$ is the mutational variance-covariance matrix (assuming that mutational effect size is drawn from an uncorrelated bivariate normal distribution with very small standard deviation *σ*). At evolutionary equilibrium *z*^⋆^, the selection gradient vanishes, $D(z^{⋆})=0$ and directional evolution comes to a halt.

Because the payoff of the joint games is simply the sum of the individual games, each element of the selection gradient vector is equal to the selection gradient of the corresponding one-dimensional csg, $D_{i}\left(z\right)=D\left(z_{i}\right)=B^{\prime }\left(2z_{i}\right)-C^{\prime }\left(z_{i}\right)$. Accordingly, when the cost and benefit functions are equal for all games, the approach to the equilibrium proceeds along a straight line. The equilibrium for each trait equals the equilibrium of the one-dimensional csg, derived in [25]. With our choice of quadratic cost and benefit function (section 2.1), there is a single evolutionary equilibrium for each public good, at $z_{i}^{⋆}=\frac{a-c}{4ab-2cd}$.

Generally speaking, the full stability analysis for a multi-dimensional model such as ours requires calculating and analysing the Jacobian matrix (convergence stability requires that the real parts of all eigenvalues are negative) and the Hessian matrix (evolutionary stability requires that the real parts of all eigenvalues are negative), see [31]. However, because in our model the total payoff is simply given by the sum of the payoffs associated with each public good, both the Jacobian and the Hessian are diagonal matrices, with the nonzero elements being the quantities required to calculate stability along each individual game. Hence, the equilibrium is an attractor of the evolutionary dynamics (i.e., convergent stable) along dimension *i* provided that $\text{d}D/\text{d}z_{i}{|}_{z_{i}=z_{i}^{⋆}}=2B^{\prime \prime }\left(2z_{i}^{⋆}\right)-C^{\prime \prime }\left(z_{i}^{⋆}\right)<0$. If this is the case, then the population converges to the equilibrium, but its subsequent evolutionary fate depends on whether *z*^⋆^ is a maximum or a minimum of the fitness landscape along each dimension *i*. If it is a maximum, $\partial^{2}/{\left(\partial z_{i}^{\prime }\right)}^{2}w(z_{i}^{\prime },z_{i}^{⋆}){|}_{z_{i}=z_{i}^{⋆}}=B^{\prime \prime }\left(2z_{i}^{⋆}\right)-C^{\prime \prime }\left(z_{i}^{⋆}\right)<0$, then the equilibrium is evolutionarily stable (meaning that it is an endpoint of evolution). If it is a minimum, then branching occurs. These conditions are identical to those given in [25]. Since we are interested in studying multidimensional evolutionary branching, we chose parameters for which the equilibrium is convergent stable but evolutionarily unstable, which is the case whenever ^*cd*^/_2*b*_ < *a* < ^*cd*^/_*b*_; in all figures, we use the values *a* = 1, *b* = 1.05, *c* = 0.9, and *d* = 1.65. Then, evolutionary branching causes the population to divide into multiple clusters, which evolve toward the borders of the phenotype space. Since the phenotype space is two-dimensional, no more than three such clusters may result from the initial branching event [32], but subsequent branching events may occur.

**Fig 1:**
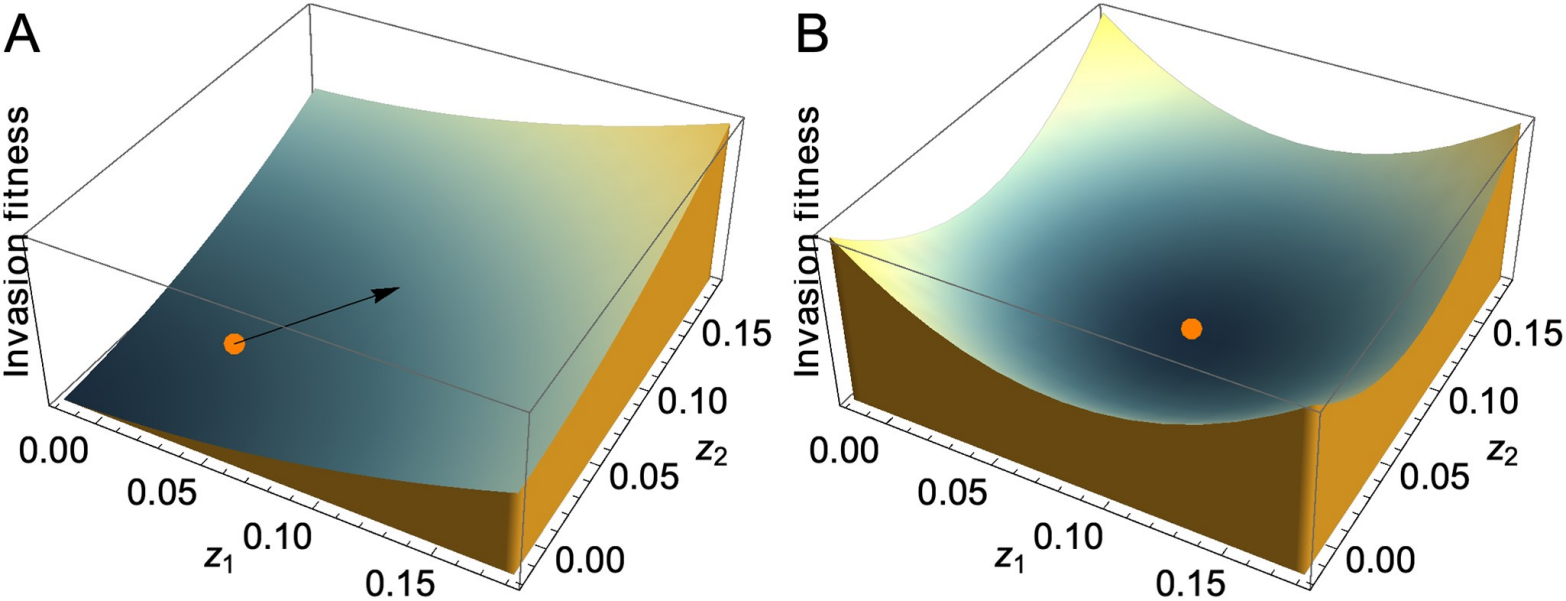
Fitness landscape during approach to the branching point (A) and at the branching point (B). **A**: When the resident (orange dot) is away from the branching point (tip of arrowhead), it moves up the fitness landscape. **B**: When the resident (orange dot) is at the branching point, the fitness landscape in our model is symmetric about the resident: all mutants equidistant to the resident have equal invasion fitness. Hence, invasion fitness alone does not predict any particular direction of branching.

Once branching has occurred and the resident population has been replaced by multiple resident branches, it is possible to use adaptive dynamics to study the fate of those branches; however, using adaptive dynamics alone, it is not possible to predict the direction of branching (although we can make a prediction using the concept of the evolutionary branching line, [14]; see Discussion and Appendix D in S1 Text citation). In fact, since the fitness landscape is entirely symmetric about the evolutionary branching point, it follows that at evolutionary branching point all equidistant mutants have the same fitness (Fig 1). In other words, adaptive dynamics does not suggest any particular direction of branching.

To understand the most likely direction of branching, we turned to computer simulations.

### 2.3 Simulations

In order to study the branching process, we perform three different kinds of simulations (explained below), of which two are stochastic (individual-based simulation and oligomorphic stochastic simulation) and one is deterministic (partial differential equation numerical simulation), and we calculate the direction of branching in these simulations. The reason for choosing these three methods is that the individual-based and the partial differential simulations explicitly track the population’s phenotypic distribution, whereas the oligomorphic simulation ignores this information. For the stochastic simulations, we focus on the most frequent outcome where the population divides into two branches and discard replicates from our analyses that contained more than two branches at the end of the initial branching event (this is the reason why some of the figures do not have a round number of replicates). Two-way branching is the most common mode of adaptive diversification [33], and branching into more than three clusters is not possible in a two-dimensional phenotype space [32]. However, subsequent branching events sometimes occur in our model after the initial separation into two strains, leading to transient cases with three or even four coexisting branches, that resolve back into dimorphisms after local extinction. The number of branches was determined using a density-based spatial clustering algorithm [34].

#### 2.3.1 Individual-based simulations (ibs)

Individual-based simulations, henceforth also ibs, are the most direct implementation of the birth–death process that underlies the evolutionary process. The differential equations from adaptive dynamics, as well as the other types of simulations we discuss in this manuscript, can therefore be seen as approximations or idealizations of this individual-based process. We simulated a population with a fixed size *n*, following an algorithm adapted from [25], in which interactions are modelled explicitly. When individuals reproduce, their offspring’s trait values are drawn from a normal distribution centered at the parent’s value, with a small standard deviation *σ*. For more details about the algorithm and parameters, see Appendix A in S1 Text. The interested reader can also run individual-based simulations in a web application available at: https://tinyurl.com/TwoGamesSimulation [35].

#### 2.3.2 Oligomorphic stochastic simulations (oss)

In contrast to the ibs, which exactly implements the birth–death process, the oligomorphic stochastic simulation (oss) approximates this process under the assumption that the timescales of population dynamics and evolutionary dynamics can be separated [36]. The underlying stochastic process (of which adaptive dynamics is a deterministic approximation) is a directed random walk called ‘trait-substitution sequence’ [4, 37]. At any given moment of this random walk, the population consists of a single (or a few) monomorphic phenotypes at population dynamical equilibrium (with constant total population size, *n*). Thus (in contrast to the ibs, where each strain consists of a cloud of points in phenotype space) each strain consists of a single point. A step of the random walk consists of the introduction (with some probability distribution) of successful mutant phenotypes that replace (or, in the case of evolutionary branching, coexist with) their ancestors. The probability of mutation at birth is *μ* and the mutational standard deviation is *σ*. Thus, the oss is an extension of the monomorphic stochastic model [30, 30, 38] that allows for evolutionary branching [36]. For parameters and details about the algorithm, adapted from [36], see Appendix B in S1 Text.

#### 2.3.3 Numerical simulation of partial differential equations (pde)

While adaptive dynamics provides a deterministic description of the evolving populations, it approximates each strain as a single monomorphic phenotype. Alternatively, we can describe the dynamics of the entire population distribution in phenotype space (in which case branching is the formation of multiple modes in this distribution). This results in a deterministic system of partial differential equations that corresponds to a large-population limit of the individual-based model [39], often referred to as a pde model. In order to numerically solve this system of equations, we discretize the phenotype distribution, resulting in a system of coupled ordinary differential equations. Each combination of phenotype values, 〈*z*_1_, *z*_2_〉, is referred to as a phenotype class. The population density of each class changes over time deterministically, with the rate of change depending both on natural selection and on the flow of mutations in and out of the class. Thus, we can track the entire population distribution over time. For details about the algorithm and parameters, see Appendix C in S1 Text.

### 2.4 G-matrix orientation and direction of branching

In the following sections, we summarize our results by focusing on three angles: the direction of approach, the **G**-matrix orientation (in the ibs), and the direction of branching.

#### Direction of approach

The direction of approach is the angle between the *z*_1_ axis and the line defined by the initial position and the branching point.

#### G-matrix orientation

In the ibs, the population’s phenotypic distribution can be summarized by the **G**-matrix, i.e., the variance-covariance matrix of the population’s phenotype (or more rigorously, but in our case identically, its additive genetic component). The phenotypic distribution will be longest along the direction given by the leading eigenvector of the **G**-matrix. This direction has been called the “genetic line of least resistance” [40] or the “**G**-matrix orientation” [18], and (for the two-dimensional case) we report it as the angle, in radians, between the eigenvector and the positive direction of the *z*_1_ axis. For example, when the longest extent of trait variance occurs along the *z*_2_ direction, the population will have an orientation of *π*/2; a population with strong positive covariance will have an orientation of *π*/4; and strong negative covariance corresponds to 3*π*/4. Finally, when the longest extent of variance occurs along the *z*_1_ direction, the population’s orientation is 0 (note that the **G**-matrix orientation is a periodic quantity, i.e., values close to *π* are also close to 0).

#### Direction of branching in stochastic simulations

**Fig 2:**
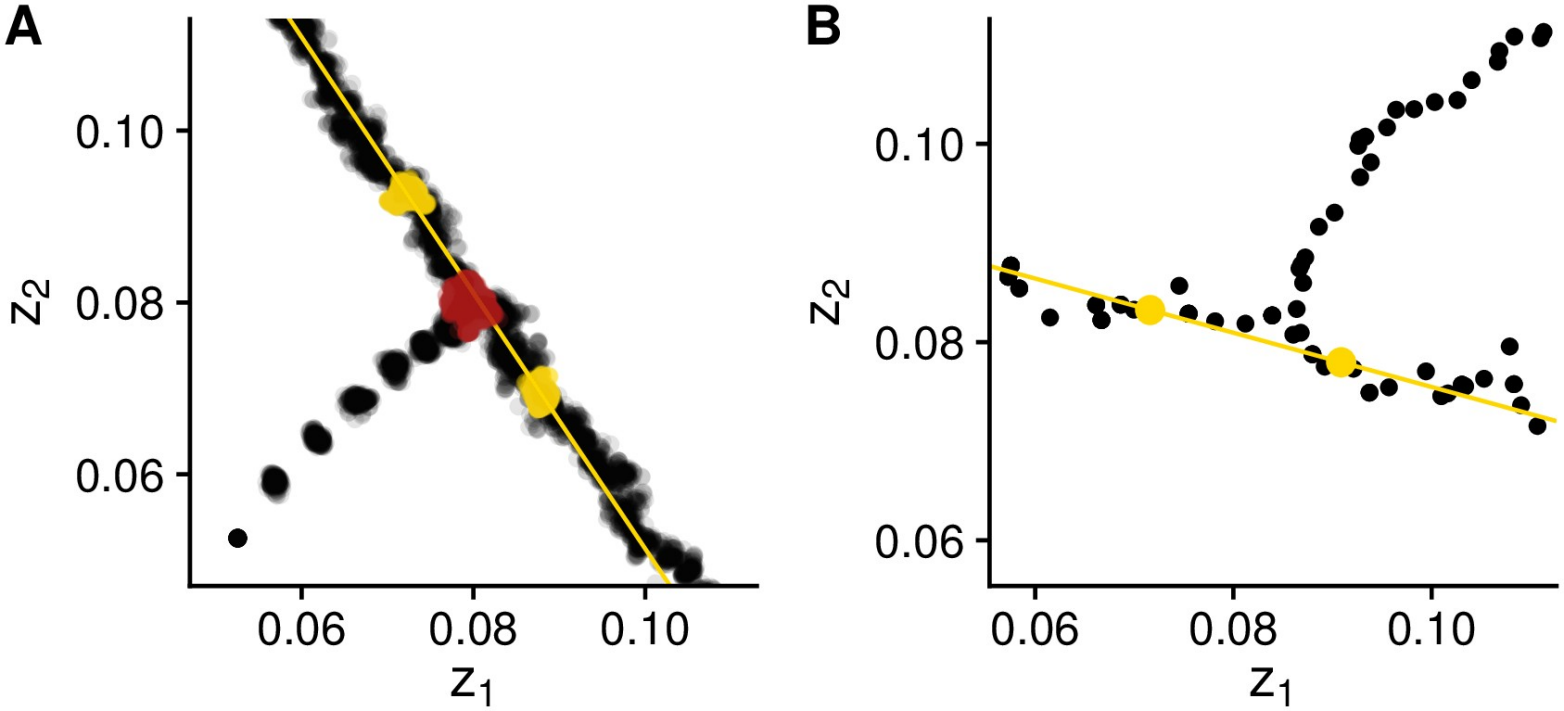
Examples of stochastic replicates of the individual-based (A) and oligomorphic (B) simulations. At each time point in the ibs, the population consists of many individuals forming a cloud in phenotype space, whereas in the oss, the population consists of a single or a few monomorphic strains. The population starts at some initial position and approaches the branching point before branching into two separate strains. The direction of approach (i.e., the angle between the *z*_1_ axis and the line defined by the initial position and the branching point) is −3*π*/4 in **A**, and *π*/4 in **B**. In **A**, dark red points indicate the *onset of branching*. In **A** and **B**, yellow points indicate the point at which we determine branching to be *complete* (and the angle between the yellow line and the *z*_1_ axis is the branching direction). Parameters: *n* = 10, 000; *σ* = 2.5 × 10^−5^ (ibs) or 1.5 × 10^−3^ (oss); *μ* = 10^−2^; for more parameters and details see Appendices A and B in S1 Text.

In all stochastic simulations, we use the same calculation that we used to calculate the **G**-matrix orientation to determine the direction of branching. We differentiate the *onset* of branching from the time point at which we consider branching to be *complete*. At the onset of branching, the population is often still monomorphic; the process is complete when the population consists of multiple, well-differentiated strains moving in different directions (for examples, see Fig 2). In our simulations, the onset of branching can be measured as the time point at which the variance in fitness is at its lowest (dark red points in Fig 2A). (When measuring the onset of branching, we exclude the first generation, in which the population has not yet accumulated variation by mutation. Note that this is not meant as a definition but simply as an operational criterion). In the ibs, we consider branching to be complete when the population’s bimodality (measured by Hartigan’s dip statistic, hds, [41]) is sufficiently high (hds > 0.15); in the oss, we consider branching to be complete when the population remains polymorphic for ten consecutive successful mutant invasions. It is at this time point that we measure the direction of branching (yellow points and line in Fig 2).

#### Direction of branching in deterministic simulations

In the deterministic simulations, branching was considered to be complete whenever any of the population’s marginal distributions (i.e., the phenotypic distribution along each of the coordinates) turned bimodal. This was considered to have occurred whenever the local maxima of the marginal distribution were at least ten times larger than the minimum in between them. The direction of branching was calculated as the angle between the *z*_1_ axis and the line passing through the local maxima of each branch.

## 3 Results

### 3.1 Perpendicular branching

**Fig 3:**
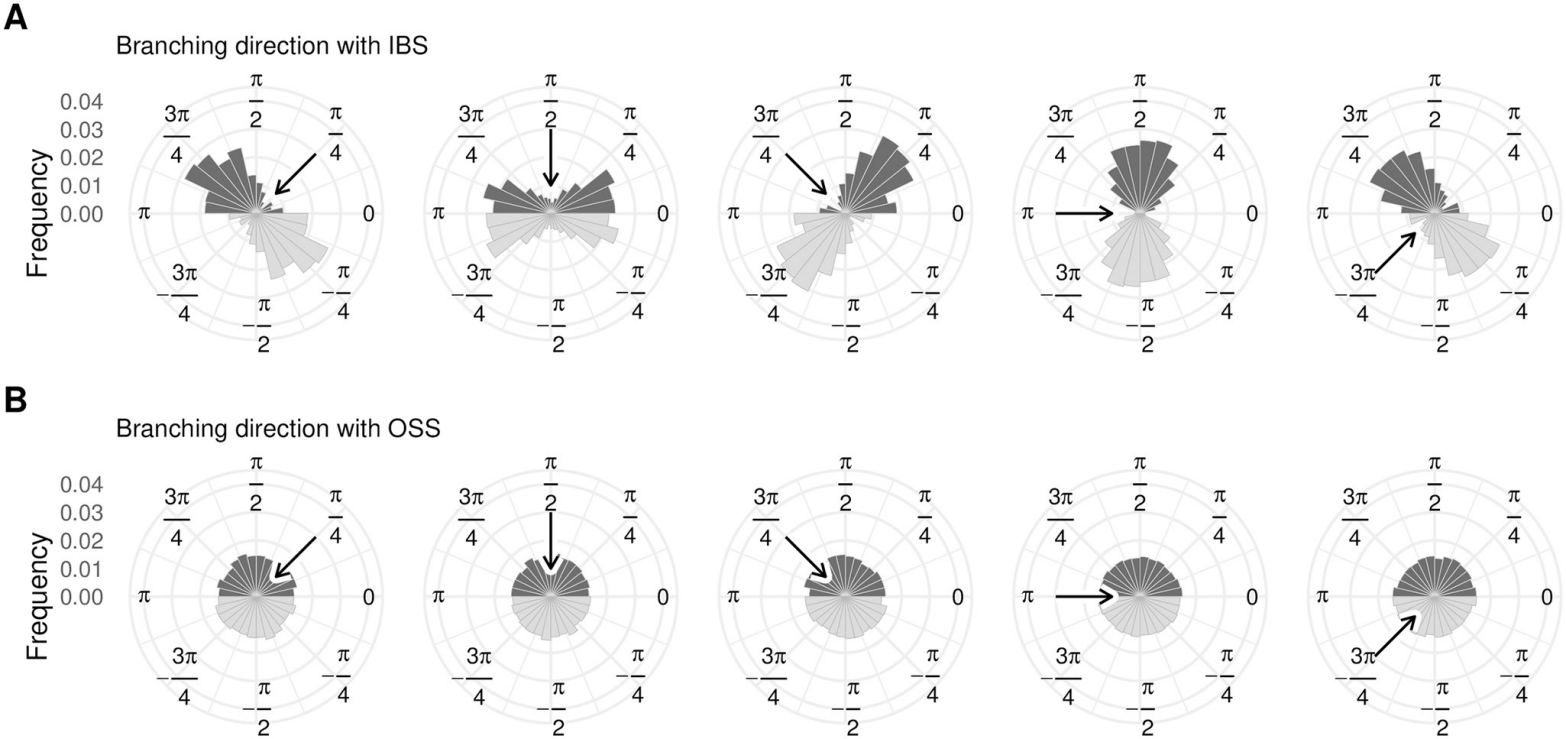
In ibs, branching tends to occur perpendicularly to the direction of approach (A), but in oss, all directions of branching are equally likely (B). Each dark histogram shows the direction of branching for 600 (in **A**) or 8,500 (in **B**) replicates. The light histogram is a mirror copy of the same data and is included for ease of visual interpretation. The arrows show the direction of approach to the branching point (from left to right: *π*/4, *π*/2, 3*π*/4, *π*, and −3*π*/4). Parameters: *n* = 10, 000; *σ* = 2.5 × 10^−5^ (ibs) or 1.5 × 10^−3^ (oss); *μ* = 10^−2^; for more parameters and details see Appendices A and B in S1 Text.

Since the two public goods are interchangeable, it follows that when the resident population is at the branching point the fitness landscape is perfectly symmetric (Fig 1). As discussed in section 2.2, this implies that, following the framework of adaptive dynamics, all directions of branching should be equally likely. However, results from individual-based simulations (section 2.3.1) show that this is not the case. Instead, branching tends to occur along the direction that is orthogonal to the direction of approach (Fig 3A).

Why is the distribution of branching directions not uniform? One possibility is that, by reducing the population to a single, monomorphic point in phenotype space, adaptive dynamics discards information that is necessary for predicting the most likely direction of branching. Real populations consist of many individuals with slightly different phenotypes, and it is possible that the population’s distribution in phenotype space depends on the direction of approach to branching point. The strength of natural selection increases with variance in fitness [42]. Hence, at the branching point, disruptive selection will tend to be strongest along the direction of highest variance (i.e., the **G**-matrix orientation), and, all else being equal, we would expect branching to occur along that direction.

To investigate this hypothesis, we implemented two alternative simulation methods: (*i*) an oligomorphic stochastic simulation (section 2.3.2), in which each strain is monomorphic; and (*ii*) a partial differential equation model (section 2.3.3), in which the entire phenotypic distribution is deterministically simulated.

**Fig 4:**
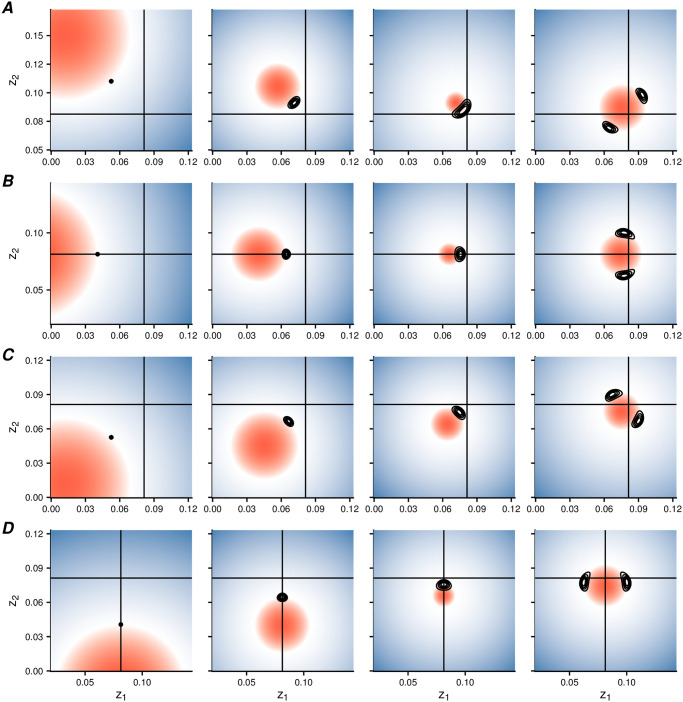
In the pde model, the population branches perpendicularly to the direction of approach. Each row shows four illustrative time points (with time moving left to right) of a pde numerical simulation. The population (whose phenotypic distribution is shown by the contour lines) approaches the branching point (intersection of the black lines) from a given direction (**A**: 3*π*/4, **B**: *π*, **C**: −3*π*/4, **D**: −*π*/2), and then branches. Colors indicate invasion fitness (red: negative values; blue: positive values; white: zero). For more details and parameter values see Appendix C in S1 Text.

Fig 3B shows that in the oligomorphic stochastic simulation (which simulates a trait-substitution sequence, section 2.3.2), all branching directions are equally probable. This conclusion corresponds to the prediction of adaptive dynamics. In contrast to the oss results, when we implement a partial differential equation (pde) numerical simulation (which deterministically tracks the entire phenotype distribution, section 2.3.3), it shows the population branching in a direction that is perpendicular to the direction of approach (e.g., Fig 4), similar to the ibs.

The results from the pde and oss simulations are in agreement in our hypothesis that the population’s phenotypic distribution plays a role in determining the direction of branching. To further investigate this effect, we now investigate how this distribution changes during the approach to the branching point and its relation to the branching direction.

### 3.2 Changes in the G-matrix orientation underlie the direction of branching

Our hypothesis is that during the approach to the evolutionary branching point, the population’s phenotypic distribution changes. The low-fitness individuals at the tail end of the traveling wave are constantly eliminated by natural selection. Simultaneously, because individuals on any given isocline of the fitness landscape are not under selection relative to each other, drift spreads the population outwards along the isoclines’ direction. Hence, we hypothesize that as the population approaches the branching point, its **G**-matrix becomes oriented perpendicularly to the direction of approach, which is consistent to the pde observations (Fig 4) and with previous theoretical work on directional selection [29].

This hypothesis is in line with the long-standing quantitative genetics result according to which, if we approximate the population’s distribution as bivariate normal, the **G**-matrix after selection is given by $G^{*}=G\left(\gamma -DD^{T}\right)G$ [15, 43, 44]. Here, ***γ*** is a matrix describing the curvature and orientation of the adaptive landscape, *γ*_*i*,*j*_ = ∂^2^*w*/(∂*z*_*i*_∂*z*_*j*_), and D is again the vector of selection gradients (Eq 1; this is often called *β* in the quantitative genetics literature). Imagine what happens to a population whose phenotypic distribution is initially perfectly symmetric, with some additive genetic variance *V* along both axes and no covariance (which matches the initial condition in our system, where the population begins as an uncorrelated bivariate normal). For simplicity, assume that the population lies along the identity line (*z*_1_ = *z*_2_), such that $D_{1}=D_{2}=D$ (for the general case we could use a rotated coordinate system, similar to the one used in Appendix D in S1 Text). Furthermore, *γ*_11_ = *γ*_22_ = Γ, and *γ*_21_ = *γ*_12_ = 0. Hence, one round of selection results in the following variance-covariance matrix:

$$\begin{array}{c} G^{*}=[\begin{array}{cc} V+\left(\Gamma -2D^{2}\right)V^{2} & -2D^{2}V^{2}\\ -2D^{2}V^{2} & V+\left(\Gamma -2D^{2}\right)V^{2} \end{array}], \end{array}$$ (2)

which has a negative covariance. Because the selection gradient has the same direction as the identity line, this buildup of negative covariance implies that the distribution becomes elongated with the axis of minor variation matching the direction of the selection gradient (as in Fig 4).

**Fig 5:**
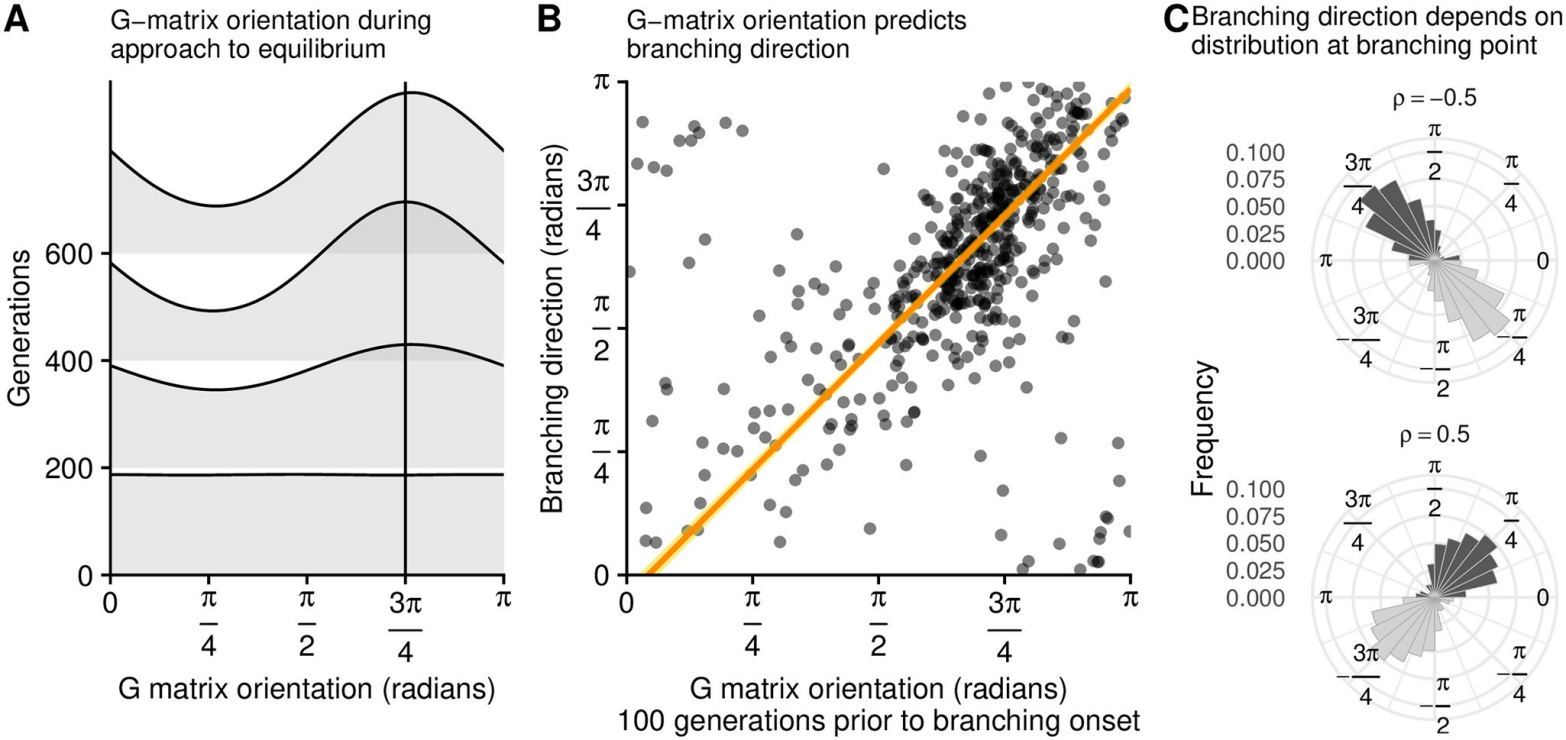
ibs results show that changes in the G-matrix orientation (A) underlie the direction of branching (B, C). Panels **A** and **B** correspond to 500 ibs replicates approaching the branching point from direction −3*π*/4 (i.e., from the bottom-left of the phenotype space). **A**: as the population approaches the branching point, the population’s phenotypic distribution becomes elongated along the direction that is perpendicular to movement. (In this case, the **G**-matrix orientation distribution becomes peaked at 3*π*/4, consistent with the second and third panels in Fig 4C.) **B**: Although there is high stochasticity, the **G**-matrix orientation prior to the onset of branching predicts the eventual direction of branching. The orange line and yellow confidence bands result from a linear regression modified to take into account the circular variable space. **C**: All else being equal, different **G**-matrix orientations cause different directions of branching. Each dark histogram shows the branching direction of 600 ibs replicates initialized at the branching point, with individuals drawn from a bivariate normal distribution with a positive (top) or negative (bottom) Pearson correlation coefficient (*ρ*). The light histograms are a mirror copy of the same data and are included for ease of visual interpretation. Parameters: *n* = 10, 000; *σ* = 2.5 × 10^−5^; for more parameters and details see Appendix A in S1 Text.

To test this hypothesis, we measure the **G**-matrix orientation in the ibs during approach to the branching point. We find that, consistent with our expectation, the population’s distribution (which starts out symmetrical) rapidly becomes perpendicular to the direction of approach (Fig 5A).

If this change in phenotypic distribution indeed explains the pattern we observe, then the **G**-matrix orientation prior to branching should be a predictor of the branching direction. We measure the **G**-matrix orientation 100 generations prior to the onset of branching, and find that it predicts the direction of branching (Fig 5B).

While these measurements show that the **G**-matrix orientation correlates with the direction of branching, they do not demonstrate a causal connection between the two quantities. To show that there is a causal relationship between the **G**-matrix orientation and the direction of branching, we initiated ibs at the branching point, with individuals drawn from bivariate normal distributions with a Pearson correlation coefficient of either *ρ* = 0.5 (**G**-matrix orientation: *π*/4) or *ρ* = −0.5 (**G**-matrix orientation: 3*π*/4). Consistent with our predictions, the most common direction of branching matches the direction of highest variance (Fig 5C). Naturally, this follows from the fact that the population’s mean trait is at the branching point (which is a fitness minimum), so that the phenotypes further away have a higher fitness.

### 3.3 Effect of the branching direction in more than two dimensions

**Fig 6:**
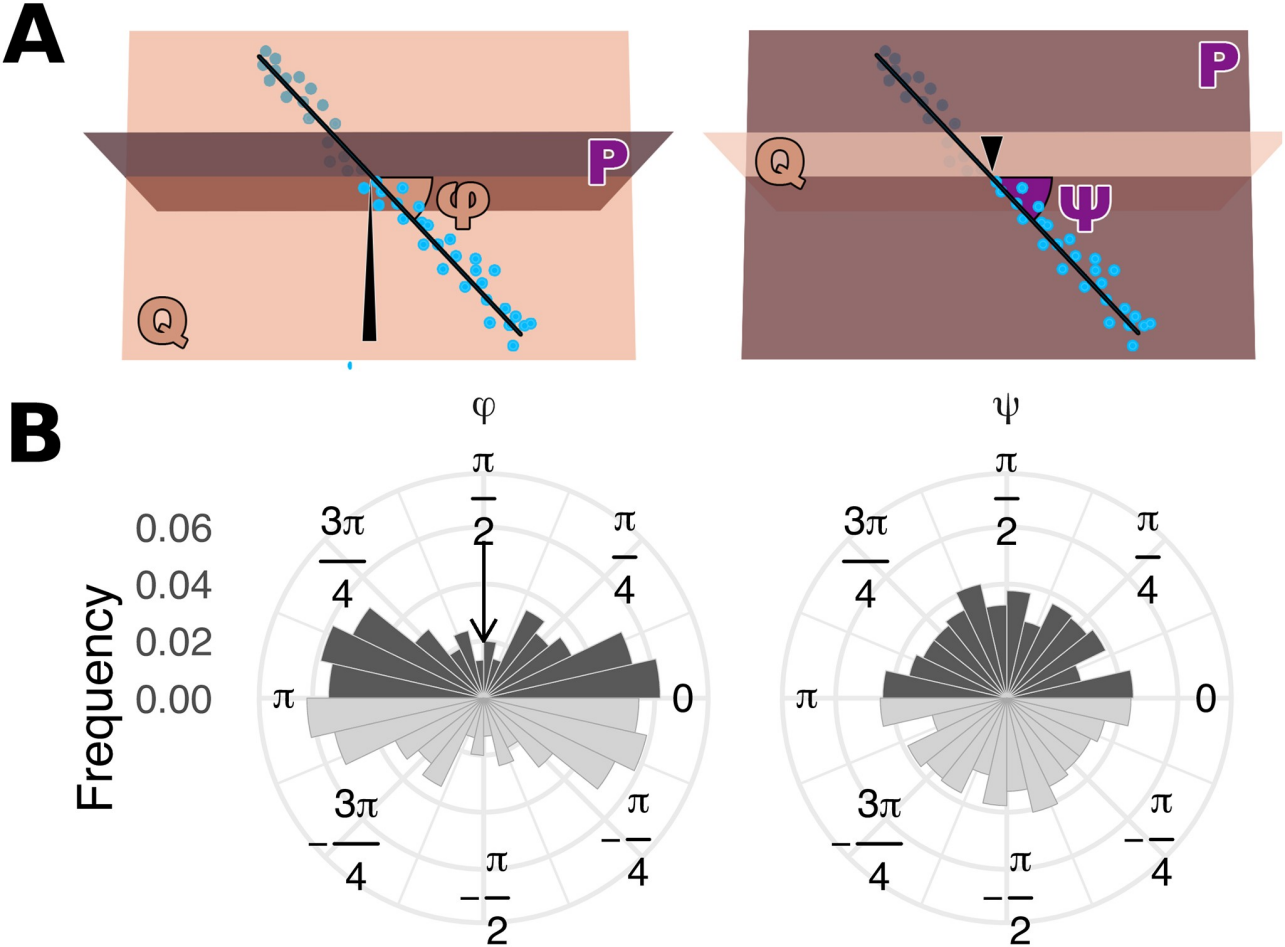
Branching in three dimensions also tends to be perpendicular to the direction of approach. We quantify direction of branching in three dimensions with two angles, *φ* and *ψ*. **A**: cartoons illustrating the interpretation of these angles (see text for details). The black arrowheads indicate the direction of approach, such that *φ* belongs to the same plane as the direction of approach (plane *Q*) and *ψ* belongs to a plane perpendicular to the direction of approach (plane *P*). The points show the population’s evolution after branching (bright blue points are above the plane and dark blue points are below it). **B**: the dark histograms show *φ* and *ψ* for 597 replicates (the light histograms are a mirror copy of the same data and are included for ease of visual interpretation). In the left-side histogram, the arrow illustrates the direction of approach; in the right-side histogram, the direction of approach is perpendicular to the circular coordinate plane. Whereas *ψ* has a uniform distribution, the frequency of *φ* peaks at 0 and *π*, meaning that branching tends to occur along a plane that is perpendicular to the direction of approach. Parameters: *n* = 10, 000; *σ* = 2.5 × 10^−5^; for more parameters and details see Appendix A in S1 Text.

The result that branching occurs perpendicularly to the direction of approach holds also for ibs in higher dimensions, i.e., when agents are engaged in three simultaneous games. To quantify the direction of branching in the three-dimensional system, we consider two perpendicular planes, *P* and *Q* (Fig 6A), whose intersection we call *I*. Plane *Q* includes the initial position $\langle z_{1}^{0},z_{2}^{0},z_{3}^{0}\rangle$, the equilibrium, and an arbitrary point 〈1, 2, 3〉. In other words, plane *Q* includes the direction of approach, i.e., the line defined by the initial point and the equilibrium point (black arrowhead in Fig 6A). Plane *P*, in turn, is perpendicular to the direction of approach and it also includes the equilibrium. Then, let the projection of *L* (the line defined by the mean phenotypes of both strains, represented in black in Fig 6A) on *P* be called *L*_*P*_ and the projection of *L* on *Q* be called *L*_*Q*_. We characterize the direction of branching using two angles. The first, *φ*, is the angle between *I* and *L*_*Q*_. The second, *ψ*, is the angle between *I* and *L*_*P*_. We find that *φ* tends to be close to 0 or *π*, meaning that branching occurs perpendicularly to the direction of approach. In contrast, all values of *ψ* are equally likely (Fig 6B), which is in agreement with our hypothesis, since all lines in *P* are perpendicular to the direction of approach.

### 3.4 Effect of the direction of approach for unequal games

We have shown that, when the two games are interchangeable, the direction of approach determines the most probable direction of evolutionary branching. While interchangeable games are not inconceivable (e.g., as the result of gene duplications, [45]), an arguably more likely scenario is that the two public goods have different fitness effects. We can quantify this difference by an asymmetry parameter *α*, such that $P\left(z^{\prime },z\right)=\left(1-\alpha \right)P_{1}\left(z_{1}^{\prime },z_{1}\right)+\alpha P_{2}\left(z_{2}^{\prime },z_{2}\right)$, i.e., the weight of the *z*_2_ payoff on fitness is *α*. When *α* = 0.5, the two games are identical; when *α* > 0.5, good *z*_2_ contributes more to fitness than good *z*_1_; and when *α* < 0.5, the opposite is true.

**Fig 7:**
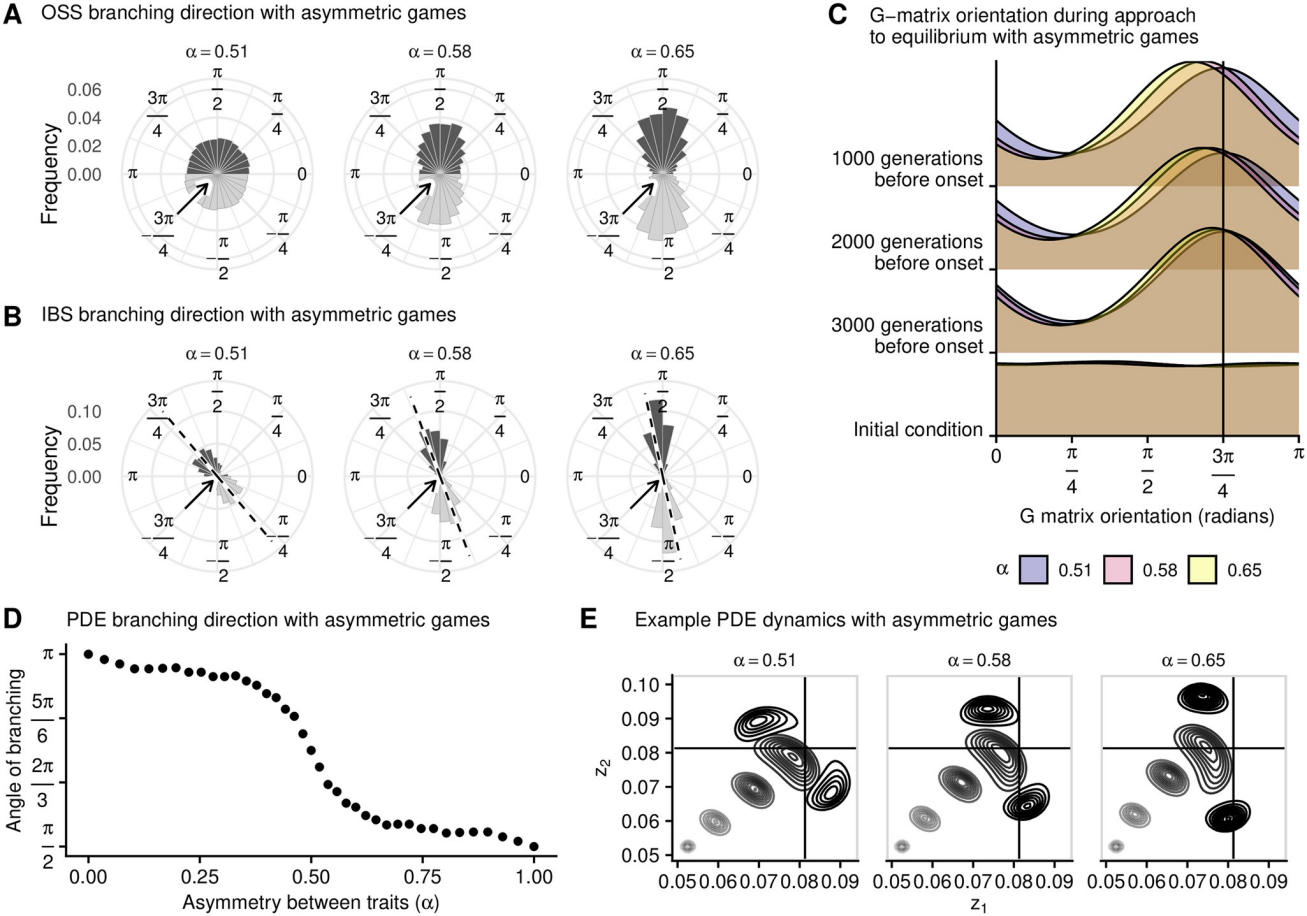
Even when games have different fitness effects, the direction of approach plays a role in determining the direction of branching. In all panels, the direction of approach to the branching point is −3*π*/4 (i.e., from the bottom-left of the phenotype space; indicated with arrows in panels **A** and **B**) and *α* is the weight of game *z*_2_ when calculating fitness. **A**: Each dark histogram shows the branching direction in oss for 7,950 replicates (light histograms are a mirror copy of the same data and are included for ease of visual interpretation). Whenever *α* > 0.5, branching tends to occur along the *z*_2_ direction (*π*/2); the distribution of branching directions becomes more peaked as *α* increases. **B**: Each dark histogram shows the branching direction for 600 ibs replicates (light histograms are a mirror copy of the same data and are included for ease of visual interpretation). As *α* increases, the most common direction of branching gradually changes from 3*π*/4 (perpendicular to direction of approach) to *π*/2. The dashed lines are the pde predictions. **C**: Frequency distribution of **G**-matrix orientation through time, as the population approaches the branching point, for different values of *α*. **D**: Branching direction for pde simulations, for increasing values of *α*, showing that the direction of approach has an effect on the branching direction even for relatively high values of asymmetry. **E**: Example pde numerical simulations with three different values of *α*. In each panel, contour lines show the population’s phenotypic distribution at different points in time. Parameters: *n* = 10, 000; *σ* = 2.5 × 10^−5^ (ibs) or 1.5 × 10^−3^ (oss); *μ* = 10^−2^; for more parameters and details see Appendices A, B and C in S1 Text.

The adaptive dynamics expectation is that for any *α* > 0.5 (*α* < 0.5), evolutionary branching is most likely to happen along the *z*_2_ (*z*_1_) direction. oss simulations confirm this expectation and, furthermore, show that the larger the inequality between the games, the less stochastic the direction of branching becomes (Fig 7A). ibs simulations show that the preferred direction of evolutionary branching is still influenced by the direction of approach when *α* ≠ 0.5 (Fig 7B). Similar to what we found for *α* = 0.5 (Fig 5), the direction of branching is affected by the **G**-matrix orientation. Initially, the population’s phenotypic distribution becomes elongated in a direction that is perpendicular to the population’s movement (Fig 7C). Over time, as it approaches the branching point, natural selection causes the phenotypic distribution to elongate in one of the two directions, an effect that is strongest for higher values of *α* (Fig 7C).

The results of ibs simulations are in qualitative agreement with pde results, which show that the direction of approach plays a role in determining the direction of evolutionary branching even for high values of *α* (Fig 7D). Example numerical simulations for various values of *α* clarify that the direction of approach, which resembles a straight line when *α* ≈ 0.5, becomes curved as *α* increases (Fig 7E). The stochastic ibs results are close to the pde predictions (dashed line in Fig 7B).

**Fig 8:**
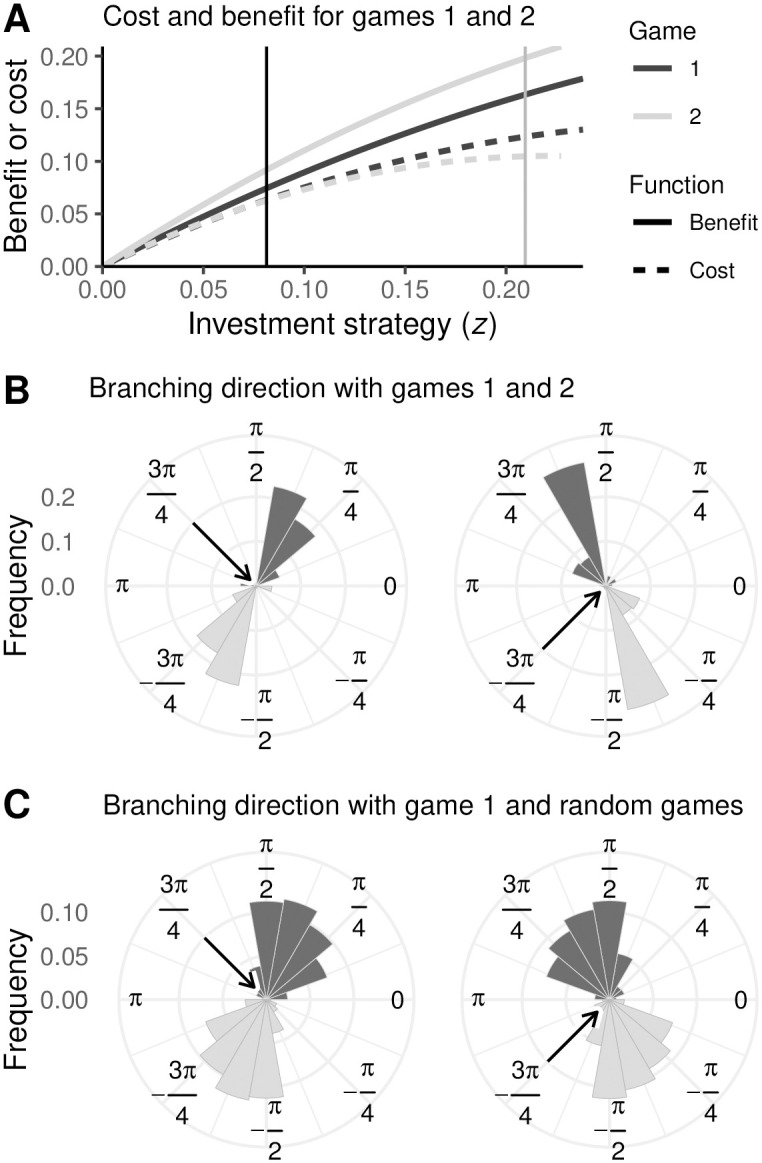
Even with different payoff (cost and benefit) functions, the direction of approach plays a role in determining the direction of branching. **A**: Example cost (dashed) and benefit (solid) functions for game 1 (black) and game 2 (grey); the respective equilibria are indicated by the vertical lines. **B**: Each dark histogram shows the ibs branching direction for 560 replicates using the payoff functions from panel **A** (light histograms are a mirror copy of the same data and are included for ease of visual interpretation). The arrows indicate the direction of approach. **C**: Each dark histogram shows the ibs branching direction for 879 replicates (light histograms are a mirror copy of the same data and are included for ease of visual interpretation). For each replicate, game 1 is the same as in panel **A** while game 2 is randomly parameterized (see text for details). The arrows indicate the direction of approach. Parameters: *n* = 10, 000; *σ* = 2.5 × 10^−5^; *μ* = 10^−2^; *a*_1_ = 1; *b*_1_ = 1.05; *c*_1_ = 0.9; *d*_1_ = 1.65. In panels **A** and **B**, *a*_2_ = 1.19; *b*_2_ = 1.1; *c*_2_ = 0.9; *d*_2_ = 2.14. For more parameters and details see Appendix A in S1 Text.

The results presented in Fig 7 show that the direction of approach still has an effect on the branching direction when the two games are the same but have different fitness effects (provided that this difference is not too large). In biologically realistic scenarios, such as the simultaneous production of different enzymes, the cost and benefit functions will not be equal for the two games. Fig 8A shows two example cost and benefit functions, which define the payoffs *P*_1_(*z*′, *z*) and *P*_2_(*z*′, *z*). These result in different selection gradients and evolutionarily stable strategies ($z_{1}^{⋆}$ and $z_{2}^{⋆}$, indicated by the vertical lines). Despite the different payoff functions, ibs results show that the direction of approach still has an effect on the branching direction (Fig 8B). This result is most pronounced when the selection gradients and the curvatures of the fitness landscapes at equilibrium are roughly similar between the two games, which in our case means that *a*_1_*b*_1_ ≈ *a*_2_*b*_2_ and *c*_1_*d*_1_ ≈ *c*_2_*d*_2_. To illustrate this, we held game 1 constant and drew random parameter values for game 2: *a*_2_ and *c*_2_ were uniformly distributed between 0 and 5; *b*_2_ and *d*_2_ were normally distributed with a mean of *a*_1_*b*_1_/*a*_2_ and *c*_1_*d*_1_/*c*_2_ (respectively) and a standard deviation of 0.1 times the mean. We then discarded any non-valid parameter combinations (i.e., combinations resulting in equilibria beyond the edges of parameter space, non–convergent stable equilibria, or evolutionarily stable equilibria). This procedure ensures that the two games are different, but have comparable selection gradients and fitness landscape curvatures around their equilibria (although the two equilibria may be very distant in phenotype space). The histograms in Fig 8C show the resulting branching directions (each replicate corresponds to a unique random parameter draw for the cost and benefit functions of game 2).

### 3.5 Eventual fate of the population

After the initial branching event, the two strains diverge toward the edges of phenotype space. Individual-based simulations reveal that, during this period of divergence, subsequent branching events (resulting in more than two strains), are common but transient. Eventually, the population will reach a stable state. Divide the phenotype space into quadrants separated by the horizontal and vertical lines that cross the equilibrium; in principle, at the end of the simulation, the population strains could occupy any combination of these quadrants. Our simulation results reveal that only two of these combinations were observed (out of 4,000 replicates). The first outcome, which we term ‘division of labor’ (dol), involves two complementary strains (each strain is a specialist that invests only into one of the goods, and not into the other good). The second outcome, which we term ‘cooperator and defector’ (cd), involves a generalist cooperator strain that invests into both goods, and a full defector that does not invest into either good.

**Fig 9:**
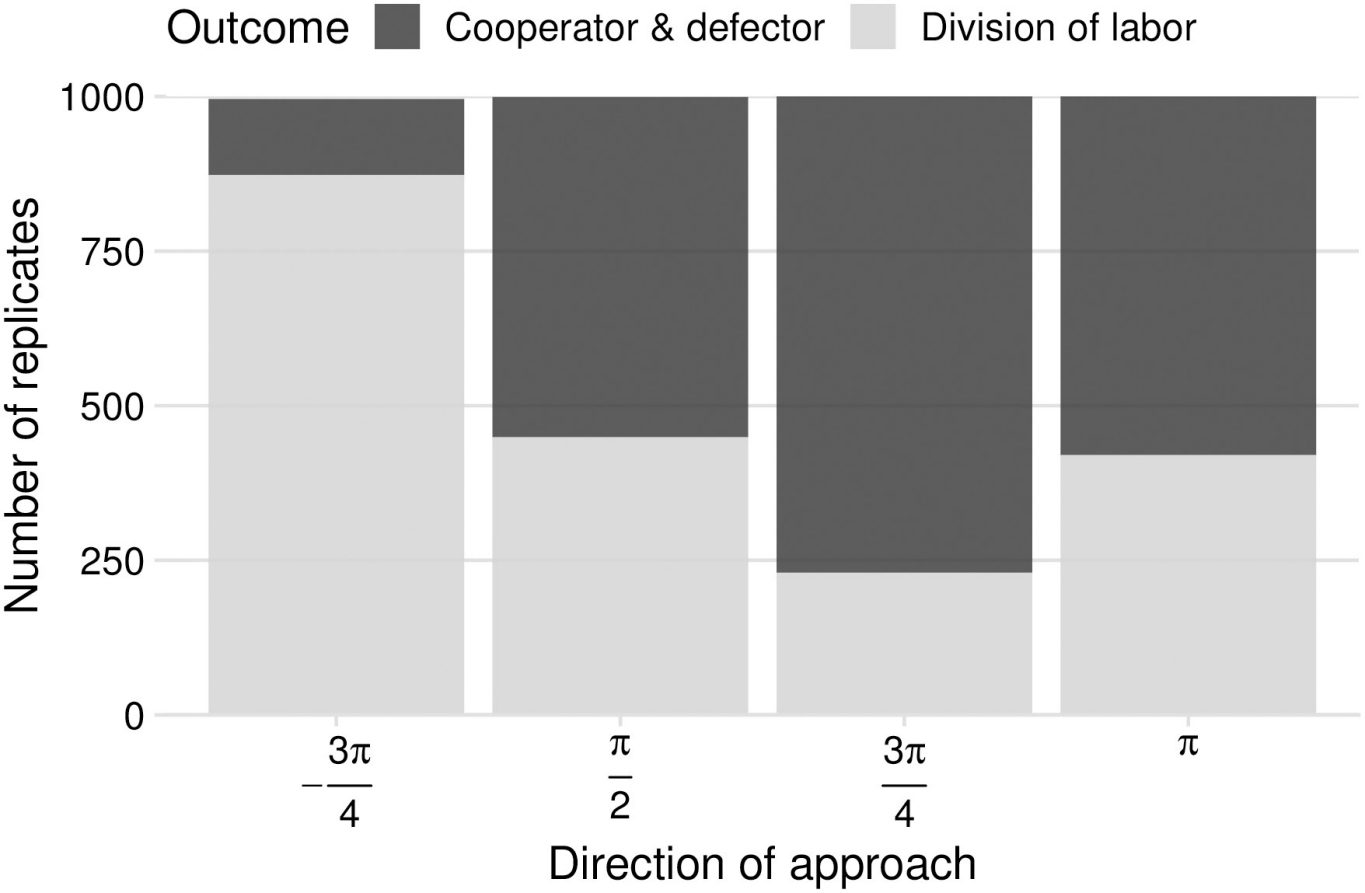
The initial direction of approach biases evolution towards one of the two possible stable states: ‘cooperator and defector’ (one strain that cooperates on both traits and one that defects on both) or ‘division of labor’ (a pair of complementary strains that cooperate on one trait and defect on the other). No other outcomes were observed. Parameters: *n* = 10, 000; *σ* = 2.5 × 10^−5^; *μ* = 10^−2^. For more parameters and details see Appendix A in S1 Text.

As a consequence of its effect on the branching direction, the angle of approach to the branching point has a very strong influence on which of the two outcomes is eventually reached. Fig 9 shows that, whenever the approach occurs along the phenotypic axes, both outcomes are roughly equally likely, but when the approach is along a diagonal direction, the outcome is determined by the most likely branching direction. Hence, the initial position of the population biases evolution towards one of the two possible stable states. This result suggests that division of labor between two traits can readily evolve without any trade-offs, whenever both of the traits evolve from a small initial value.

The two outcomes we discussed so far are only stable when mutations are small, as we assume throughout this article. In Appendix F in S1 Text, we investigate what would happen if we allowed a large-effect mutation—specifically, a mutant occupying any one of the empty corners of the phenotype space—to occur in a resident population with a cd or dol configuration. We find that while the cd configuration is stable against invasion, the dol configuration is susceptible to invasion by a third strain. The successful third strain will be a cooperator if *z*_max_/2 < *z*^⋆^, or a defector if *z*_max_/2 > *z*^⋆^ (Appendix F in S1 Text). The resulting three-strain equilibrium is stable against invasion by a fourth strain.

## 4 Discussion

Evolutionary game theory studies simplified interactions (games) between individuals. These games represent common tasks that organisms perform in nature, such as the production of an enzyme [23], competition for a mating partner [46], or participation in a hunting group [47]. Traditionally, these games are studied in isolation, and it is assumed that the resulting evolutionary dynamics are independent between games. However, in nature, the same individual will engage in many different kinds of interactions, either simultaneously or throughout their lifetime, which increases the trait space dimension and may lead to novel evolutionary outcomes that are not predicted from each game’s individual dynamics (e.g., [48–50]). Here, we have studied the case of evolutionary branching when a population is engaged in multiple continuous snowdrift games.

We have shown that there is a feedback between evolutionary dynamics and the population’s phenotypic distribution: the branching direction can be influenced by the population’s phenotypic distribution, which in turn is affected by the direction of approach to the evolutionary branching point. As a consequence, knowing that each trait undergoes branching is not sufficient to predict the direction of branching in multidimensional space.

The dynamics of multiple simultaneous games cannot be predicted from the individual games, even when their payoffs are additive. Analysis of discrete-strategy games reveals that when games have at least three pure strategies, the resulting multi-game dynamics cannot be characterized from the separate analysis of each game [27, 28, 51]. Together with these previous results, our study poses an important challenge because most of our understanding of frequency-dependent evolutionary dynamics is built on single-game studies, despite the fact that multiple simultaneous games are arguably the most common scenario.

Although we motivated our study by focusing on simultaneous continuous games, our results are, more broadly, relevant for our understanding of evolutionary branching in multidimensional spaces. As with multi-game dynamics, a growing number of studies has revealed that some properties of multidimensional evolutionary branching cannot be predicted from studying each dimension in isolation. These studies have shown that correlations between traits (caused by pleiotropy, epistasis, or linkage disequilibrium) can influence the stability of equilibria and the direction of branching [8, 10].

Similarly to our result, previous studies [11–14] have also found that branching can occur orthogonally to the selection gradient. This phenomenon has been formally studied using the concept of evolutionary branching lines [14], the idea of which is that the strength of directional selection decreases as the population approaches the branching point, and it may be overwhelmed by strong disruptive selection orthogonal to the selection gradient. Although the evolutionary branching line approach predicts the same branching direction as our model (Appendix D in S1 Text), we argue that our results are instead explained by the effect of the population distribution because of the following two reasons. First, the predictions of the evolutionary branching line can be tested by using the method of maximum likelihood invasion paths (mlip, [13]) or oligomorphic stochastic simulations. Both of these cannot predict the observed branching direction: in our system, mlip predicts branching along the approaching direction (Appendix E in S1 Text), and the oligomorphic stochastic simulations do not exhibit any preferred direction of branching (Fig 3B). These results suggest that at least in our system those effects may be very weak or easily overwhelmed by stochasticity. Second, our pde model shows that the shape of the population’s phenotypic distribution can underlie the direction of branching even under circumstances where the evolutionary branching lines would make the opposite prediction (see Fig A in Appendix D in S1 Text). Branching is more likely to occur along the axis of greater variation, which acts as a “genetic line of least resistance” for adaptive diversification [40]. Not only does the phenotypic distribution affect evolutionary dynamics, but in turn it is also affected by the direction of movement (Fig 5), creating a distribution–selection feedback that is not captured in analytical models of evolutionary branching, which assume monomorphic populations.

In order to more clearly demonstrate the importance of the phenotypic distribution, we have purposefully chosen a set of assumptions that removes any other causes for a non-uniform distribution of branching directions. Among those assumptions are: equal payoff functions, independent traits, and equal, uncorrelated mutations for each phenotype. Violating any of these would impose a preferred branching direction, even for a monomorphic population. This is illustrated in our work by relaxing the assumption that both traits are equally important for fitness. As the fitness effects become increasingly asymmetric, a particular branching direction becomes more likely even when the population is monomorphic (Fig 7A). However, our results also show that the population distribution can play a role even when this assumption is relaxed, provided that asymmetry is not too high.

For future extensions it would be interesting to expand this model to a broader universe of game-theoretic scenarios, including *N*-player groups [52], competitive interactions [50, 53], synergy and discounting [54], or the effect of spatial or social structure [55].

The branching direction has important long-term consequences because it leads the population toward different stable states. Hence, different directions of approach to equilibrium result in different outcomes. A similar phenomenon, albeit with a different mechanism, occurs in a one-locus two-alleles model by [56]. In that model, the occurrence of branching slightly before the branching point places the two strains in different basins of attraction (depending on which side of the equilibrium branching takes place). In our model, there are two alternative evolutionary outcomes: one consists of a cooperator and a defector strain, and the other consists of two specialists strains, each of which produces one public good and consumes the other (division of labor). This second outcome, which is most likely to occur whenever both evolving traits approach the branching point from the same direction, resembles the production and sharing of metabolites that occurs, for instance, between microbes in multi-species biofilms [57]. These interactions can certainly be interpreted as a (very) high-dimensional set of simultaneous games where specialization and division of labor are common. As microbes in multi-species communities specialize in particular metabolites and functions, communities may give rise to networks of mutual dependency (Black Queen evolution, [58]). Our model shows how this type of mutual dependency may evolve even in the absence of the costs and trade-offs that are often invoked in other models of cooperation-based specialization (e.g., [59]).

Our results exemplify how signatures of past selection can impact the response to current selection pressures. Models that disregard these signatures (e.g., by assuming monomorphic populations) discard information that may be relevant for predicting evolutionary trajectories.

## Supporting information

S1 Text Supporting information. Algorithms for implementing the individual-based simulation (Appendix A), the oligomorphic stochastic simulation (Appendix B) and the partial-differential equation model (Appendix C). Derivation of the condition for occurrence of branching according to the evolutionary branching line (Appendix D and Fig A) and according to the maximum-likelihood invasion path (Appendix E). Effect of the invasion of large-effect mutants (Appendix F).
(PDF)
