# Supplementary material for: On the importance of evolving phenotype distributions on evolutionary diversification
Source: PLoS Comput Biol. 2021 Feb 16;17(2):e1008733. doi: 10.1371/journal.pcbi.1008733 (PMC7909671; doi:10.1371/journal.pcbi.1008733)
Supplement: S1 Text — Algorithms for implementing the individual-based simulation (Appendix A), the oligomorphic stochastic simulation (Appendix B) and the partial-differential equation model (Appendix C). Derivation of the condition for occurrence of branching according to the evolutionary branching line (Appendix D and Fig A) and according to the maximum-likelihood invasion path (Appendix E). Effect of the invasion of large-effect mutants (Appendix F). (PDF) [file pcbi.1008733.s001.pdf]

Supporting Information: On the importance of evolving phenotype distributions on evolutionary diversification

Gil Jorge Barros Henriques[1], Koichi Ito[1], Christoph Hauert[2], and Michael Doebeli[1,2]

[1]Department of Zoology, University of British Columbia, Vancouver, British Columbia, Canada

[2]Department of Mathematics, University of British Columbia, Vancouver, British Columbia, Canada

Appendix A IBS algorithm

The population consists of a fixed number n of individuals. Each individual is defined by their investment strategies z (we focused mostly on the two-dimensional case $z = \langle z_1, z_2 \rangle$, but we also considered the three-dimensional case $z = \langle z_1, z_2, z_3 \rangle$). The population is updated asynchronously. At every time step, two randomly chosen focal individuals with phenotype z and z' interact with randomly chosen partners p and p', earning payoffs $P(z,p)$ and $P(z',p')$, respectively. The first focal individual, z, then dies, and the empty position is taken by the offspring of whichever of the two focal individuals has the highest payoff. Each element of the offspring's phenotype vector is drawn from a normal distribution, centered at the parent's phenotype value, with a small standard deviation σ. The simulation is ended whenever an individual's strategy reaches the edges of phenotype space.

For all figures, we used the following parameters: $n = 10,000$, $\sigma = 2.5 \times 10^{-5}$, and initial distance to the branching point $= z_1^\star/2$.

Appendix B OSS algorithm

This algorithm is adapted from Appendix A in Ito, Dieckmann [1]. For most of the simulation, the population consists of a single resident monomorphic strain. As the simulation unfolds, mutant strains are introduced. When this happens, there are three alternatives: (*i*) the mutant may invade and become the new resident; (*ii*) the mutant may fail to invade and be removed from the population; or (*iii*) the strains may be mutually invasible, in which case all strains will be resident (i.e., the population becomes polymorphic). Hence, although a small number of strains may coexist, each strain is monomorphic (in contrast with the IBS, where each strain resembles a cloud in phenotype space).

1. At the beginning of the simulation, the population consists of a single monomorphic resident strain with a two-dimensional phenotype. More generally, throughout the simulation, the population will consist of a list of N resident strains with two-dimensional phenotypes $Z = \langle Z_1, \cdots, Z_N \rangle$. Each entry of Z is a strain, i.e. a vector of trait values, $Z_i = \langle z_1^{(i)}, z_2^{(i)} \rangle$. The equilibrium frequency distribution $\hat{n} = \langle \hat{n}_1, \cdots, \hat{n}_N \rangle$ is proportional to $\mathbf{P}^{-1} J$ (where J is a column vector of ones and $P_{i,j}$ is the payoff of strain i upon interacting with strain j).

2. Then, an invasion attempt occurs. The mutant emerges from strain i with probability ν_i/ν, where $\nu_i = \mu\hat{n}_i$ and $\nu = \sum_{i=1}^{N} \nu_i$. The mutant phenotypes, Z_i', are normally distributed about the parental strain strategy. Time t is updated by $\Delta t = -(1/\nu)\ln\varrho$, where ϱ is uniformly distributed between 0 and 1.

3. The payoff of the mutant is given by $P(Z_i', Z) = \sum_{j=1}^{N} P(Z_i', Z_j)\hat{n}_j$. The payoff of the residents is calculated similarly: $P(Z_1', Z) = \sum_{j=1}^{N} P(Z_1', Z_j)\hat{n}_j$ (since, by definition, all residents have the same payoff, we arbitrarily pick resident Z_1 for the calculation). If the mutant's payoff is higher than the resident's, the probability that the mutant replaces the resident is given by $\mathcal{S}(Z_i', Z) = \left[P(Z_i', Z) - P(Z_1', Z)\right]/P(Z_i', Z)$, otherwise the probability is zero. To accelerate the simulation we work with a modified invasion probability, $\tilde{\mathcal{S}}(Z_i', Z) = 1 - \exp(-\chi\mathcal{S}(Z_i', Z))$, where the coefficient χ adjusts the magnitude of the fitness effect.

4. The invasion attempt is successful if $\varrho < \tilde{\mathcal{S}}(Z_i', Z)$, where ϱ again is a uniformly distributed random number between 0 and 1. To accelerate the simulation, if there have been over η unsuccessful invasions, set χ to $\chi \times \xi$. Similarly, if an invasion was successful in less than $\eta/2$ invasion attempts, set χ to χ/ξ. If mutant Z_i' was not successful, return to step 2.

5. If the mutant was successful, we test for mutual invasibility by calculating equilibrium frequency distributions, $\hat{n}' = \langle \hat{n}_1, \cdots, \hat{n}_i, \cdots, \hat{n}_N \rangle$, for a population where the mutant replaces the resident, $Z' = \langle Z_1, \cdots, Z_i', \cdots, Z_N \rangle$. If all elements of $\hat{n}'$ are larger than a threshold ε, and $\mathcal{S}(Z_i, Z') < 0$ at $\hat{n}'$, there is no mutual invasibility. The mutant takes the resident's place: replace Z_i with Z_i' and $\hat{n}$ with $\hat{n}'$.

6. Otherwise, the number of strains in the resident population, N, increases by one, and the mutant becomes a new element of Z. Calculate the new resident equilibrium frequencies, $\hat{n}$. Should any frequency fall below the threshold ε, remove that strain from the population. Begin a new iteration by returning to step 2.

For all figures, we used the following parameters: $n = \sum_{i=1}^{N} \hat{n}_i = 10{,}000$, $\mu = 10^{-2}$, $\sigma = 1.5 \times 10^{-3}$, $\varepsilon = 10^{-4}$, $\chi = 100$, $\eta = 10$, and initial distance to the branching point $= z_1^\star/2$.

Appendix C PDE algorithm

We used PDE simulations to study the two-dimensional model. First, we discretized phenotype space into a square matrix with classes of width and height h. At any point in time t, the payoff of every phenotype class $\langle z_1, z_2 \rangle$ is given by $P_{1,2} = \sum_{p,q} n_{1,2}(t) P(\langle z_1, z_2 \rangle, \langle z_p, z_q \rangle)$, where $n_{1,2}(t)$ is the population density of the phenotype class. The density of a phenotype class can change either by natural selection or due to the flow of mutations in and out of the class. Selection depends on the fitness of phenotype $\langle z_1, z_2 \rangle$, which equals $w_{1,2} = \max(sP_{1,2}, 0)$, where the parameter s characterizes the strength of selection. Mutations occur at a rate μ, and their effect size is assumed to be small, such that a mutant produced by phenotype $\langle z_1, z_2 \rangle$ must be a von Neumann neighbour of $\langle z_1, z_2 \rangle$. The von Neumann neighbourhood $\mathcal{N}$ comprises the immediate non-diagonal (cardinal) neighbouring classes. Further, the set excludes classes beyond the

boundaries of phenotype space. Together, selection and mutation allow us to update every class density according to the coupled system of equations

$$\frac{\mathrm{d}n_{1,2}(t)}{\mathrm{d}t} = \left(1 - \frac{\mu}{h^2}\right) n_{1,2}(t) w_{1,2} + \sum_{\langle p,q\rangle \in \mathcal{N}} \frac{\mu}{4h^2} n_{p,q}(t) w_{p,q} - n_{1,2}(t)\overline{w} - \frac{\zeta}{h^2}, \tag{C.1}$$

where $\overline{w}$ is the mean population fitness and $\zeta \ll 1$ is a change threshold. The change threshold partially mimics the effect of demographic stochasticity with finite population sizes, by preventing the low-density edges of the population distribution from rapidly spreading outward (densities were truncated at zero to avoid nonsensical negative values). This constrains the population distribution to a discrete number of compact clusters (strains), as opposed to a wide distribution over the entirely of the phenotype space. Consequently, ζ regulates the phenotypic variance of the strains.

For all figures, we used the following parameters: $h = z_1^\star/100$, $\mu = 0.01 \times (h/100)^2$, $s = 100$, $\zeta = 2 \times 10^{-7} \times (h/100)^2$. To numerically simulate the PDE system, we used the adaptive step-size Runge–Kutta–Fehlberg method [2, 3] with permissible absolute error = 10^{-5}, permissible relative error = 10^{-4}, error order = 4, and step order = 5.

Appendix D Branching condition along evolutionary branching line

In this section, we derive the condition for the occurrence of evolutionary branching in a directionally evolving population following Ito, Dieckmann [4]. The payoff of a mutant $z' = \langle z_1', z_2' \rangle$ in a resident population $z = \langle z_1, z_2 \rangle$ is:

$$\begin{aligned} w(z', z) &= a(1 - b(z_1' + z_1))(z_1' + z_1) - c(1 - dz_1')z_1' \\ &+ a(1 - b(z_2' + z_2))(z_2' + z_2) - c(1 - dz_2')z_2' \end{aligned} \tag{D.1}$$

The selection gradients for two traits, $\mathcal{D}_1$ and $\mathcal{D}_2$, are

$$\begin{aligned} \mathcal{D}_1 &= \left.\frac{\partial w}{\partial z_1}\right|_{z'=z} \\ &= a - c - (4ab - 2cd)z_1 \end{aligned} \tag{D.2}$$

$$\begin{aligned} \mathcal{D}_2 &= \left.\frac{\partial w}{\partial z_2}\right|_{z'=z} \\ &= a - c - (4ab - 2cd)z_2 \end{aligned} \tag{D.3}$$

respectively. Consider a new rotated coordinate system $\langle x, y \rangle$:

$$\begin{pmatrix} x \\ y \end{pmatrix} = \begin{pmatrix} \cos\theta & -\sin\theta \\ \sin\theta & \cos\theta \end{pmatrix} \begin{pmatrix} z_1 \\ z_2 \end{pmatrix} \tag{D.4}$$

The selection gradients on these new coordinate axes, $\mathcal{D}_x$ and $\mathcal{D}_y$, are

$$\begin{aligned}
\mathcal{D}_x &= \left.\frac{\partial w}{\partial x}\right|_{x'=x,y'=y} \\
&= \frac{\partial x}{\partial z_1}\mathcal{D}_1 + \frac{\partial x}{\partial z_2}\mathcal{D}_2 \\
&= (a-c)(\cos\theta - \sin\theta) - (4ab - 2cd)x \qquad \text{(D.5)} \\
\mathcal{D}_y &= \left.\frac{\partial w}{\partial y}\right|_{x'=x,y'=y} \\
&= \frac{\partial Y}{\partial z_1}\mathcal{D}_1 + \frac{\partial y}{\partial z_2}\mathcal{D}_2 \\
&= (a-c)(\sin\theta + \cos\theta) - (4ab - 2cd)y \qquad \text{(D.6)}
\end{aligned}$$

respectively. We can always find an adequate rotation angle θ^* under which $\mathcal{D}_x = 0$ for any focal population $\langle x, y\rangle$.

According to Ito, Dieckmann [4], the condition for the occurrence of evolutionary branching under directional selection (i.e., $\mathcal{D}_y > 0$) is

$$\mathcal{D}_x = 0 \qquad \text{(D.7)}$$

$$C_{xx} < 0 \qquad \text{(D.8)}$$

$$\sigma \frac{\gamma_{xx}}{|\mathcal{D}_y|} > \sqrt{2} \qquad \text{(D.9)}$$

where

$$\begin{aligned}
C_{xx} &= \frac{\partial}{\partial x}\mathcal{D}_x \\
&= -4ab + 2cd \qquad \text{(D.10)}
\end{aligned}$$

$$\begin{aligned}
\gamma_{xx} &= \left.\frac{\partial^2 w}{\partial x^2}\right|_{x'=x,y'=y} \\
&= -2ab + 2cd \qquad \text{(D.11)}
\end{aligned}$$

and σ is the mutation distribution. (D.7) is always satisfied when $\theta = \theta^*$. Additionally, because now we focus on the situation where evolutionary branching occurs at the singular point, (D.8) is also satisfied with our focal parameter values. By substituting (D.6) and (D.11), (D.9) is rewritten as

$$\sigma \frac{(-2ab + 2cd)}{|(-4ab + 2cd)(y - y^\star)|} > \sqrt{2} \qquad \text{(D.12)}$$

where $y^\star$ is the singular point, i.e.,

$$y^\star = \frac{(a-c)(\sin\theta + \cos\theta)}{4ab - 2cd}. \qquad \text{(D.13)}$$

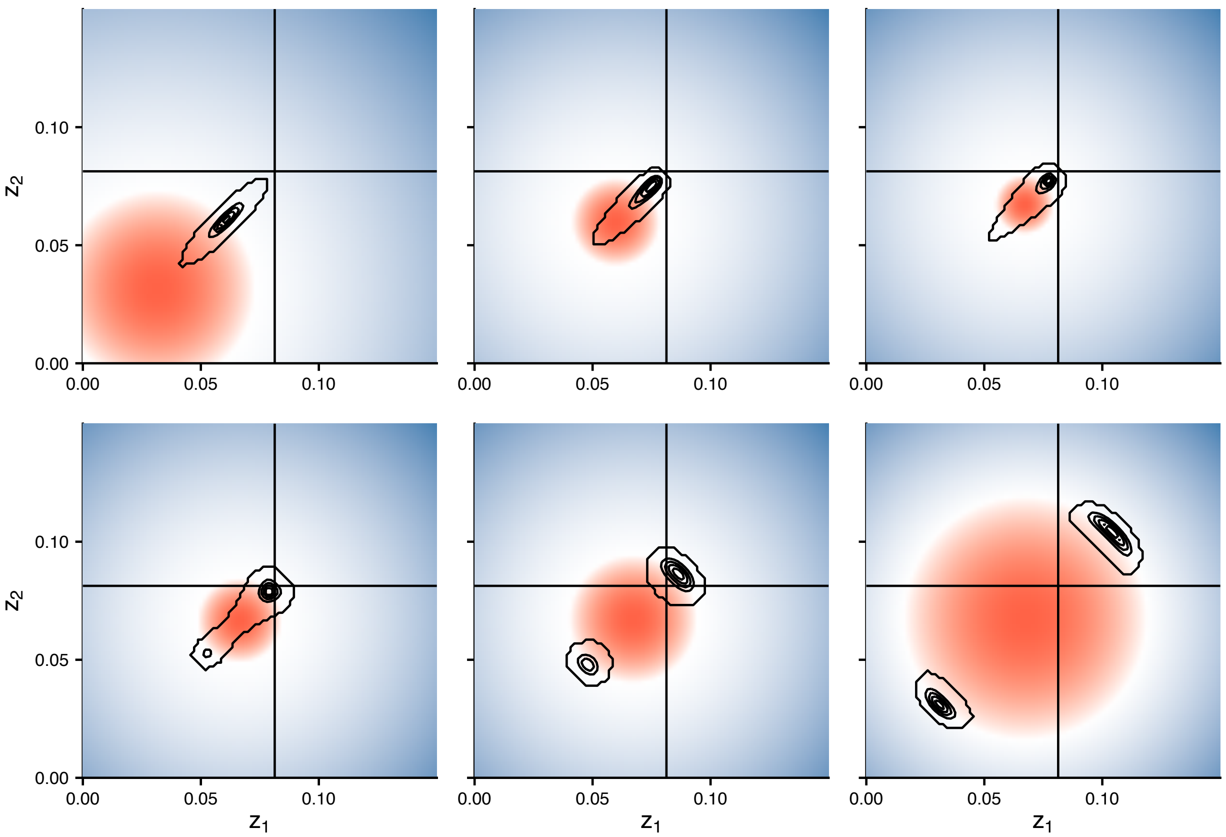

Fig A: In the PDE model, for some initial phenotypic distributions, branching may occur in directions that are different from those predicted by the evolutionary branching line approach. Each panel is an illustrative time point of a PDE numerical simulation. The phenotypic distribution is shown by the contour lines. Colors indicate invasion fitness (red: negative values; blue: positive values; white: zero). For more details and parameter values see Appendix C.

Since (D.12) is always satisfied when the resident is sufficiently close to the singular point $(x^\star, y^\star)$, evolutionary branching occurs along the x direction, i.e., orthogonal to the selection gradient [4].

Despite this prediction, partial-differential equation simulations show that, depending on the initial phenotypic distribution, branching can occur in the opposite direction (Fig A).

Appendix E Maximum-likelihood invasion path

Maximum likelihood invasion path (MLIP, [5]) is one of the methods for investigating the dynamics around the evolutionary branching line. Here, we show that the MLIP cannot predict the branching direction observed in our individual-based simulations.

Hereafter, we represent the traits using the new rotated coordinate axes from Appendix D. By using Eqs. (D.5),

(D.6), and (D.11), the payoff of a mutant $(x+\delta_x, y+\delta_y)$in a resident population (x, y) is represented as

$$P(x+\delta_x, y+\delta_y, x, y) = P(x, y, x, y) + D_x\delta_x + D_y\delta_y + \frac{1}{2}\gamma_{xx}\delta_x^2 + \frac{1}{2}\gamma_{yy}\delta_y^2 \tag{E.1}$$

By using condition (D.7) and the fact that $\gamma_{xx} = \gamma_{yy}$, the invasion fitness of the mutant, $w(x+\delta_x, y+\delta_y, x, y)$ is

$$w(x+\delta_x, y+\delta_y) = D_y\delta_y + \frac{1}{2}\gamma_{xx}(\delta_x^2 + \delta_y^2). \tag{E.2}$$

Now we assume that the mutation probability follows a two-dimensional normal distribution, i.e.,

$$M(\delta_x, \delta_y) = \frac{1}{2\pi\sigma^2}\exp\left(-\frac{1}{2\sigma^2}(\delta_x^2 + \delta_y^2)\right). \tag{E.3}$$

Then, the probability density that the focal mutant emerges and successfully invades at the next invasion event is represented as

$$\mathcal{P}(x+\delta_x, x+\delta_y) = T\mu\hat{n}M(\delta_x, \delta_y)w(x+\delta_x, y+\delta_y) \tag{E.4}$$

where T is expected waiting time for the next invasion event, μ is the probability of the mutation and $\hat{n}$ is the population size (see Eqs. (8a) and (8b) in [5]).

When we randomly choose which mutant invades following (E.4), this method is equal to the oligomorphic stochastic simulation (Appendix B). On the other hand, in the MLIP, it is assumed that the mutant which has the maximum invasion probability density will be chosen in the next invasion event. Such a mutant can be derived by investigating the extremum of Eq (E.4) for δ_x and δ_y. The mutant with maximum invasion probability density, $(x+\delta_x^*, y+\delta_y^*)$, should satisfy

$$\left.\frac{\partial\mathcal{P}}{\partial\delta_x}\right|_{\delta_x=\delta_x^*, \delta_y=\delta_y^*} = 0 \tag{E.5}$$

$$\left.\frac{\partial\mathcal{P}}{\partial\delta_y}\right|_{\delta_x=\delta_x^*, \delta_y=\delta_y^*} = 0 \tag{E.6}$$

By substituting Eq (E.2) and Eq (E.3), we can rewrite conditions (E.5) and (E.6) as

$$\delta_x^*(\gamma_{xx} - \frac{1}{\sigma}w(x+\delta_x^*, y+\delta_y^*, x, y)) = 0 \tag{E.7}$$

and

$$\delta_y^*\gamma_{xx} + D_y - \delta_y^*\frac{1}{\sigma}w(x+\delta_x^*, y+\delta_y^*, x, y) = 0 \tag{E.8}$$

respectively.

If $\delta_x^* \neq 0$, condition (E.7) is satisfied when and only when $w(x+\delta_x^*, y+\delta_y^*, x, y) = \sigma\gamma_{xx}$. By substituting this into Eq (E.8), we obtain $D_y = 0$. Because we are using a rotated coordiane system satisfying $D_x = 0$, this condition is

satisfied when and only when the population reaches the singular point; in other words, the mutant with $\delta_x^* \neq 0$ cannot invade into the resident population until the population reaches the evolutionary singular point. Consequently, under directional selection (i.e., $D_y > 0$) the MLIP always predicts the invasion of mutants whose trait value for x-axis is same as that of the resident. Because the MLIP predicts the occurrence of evolutionary branching when the invaded mutant and resident strains can coexist, the direction of branching always occurs along the y direction. This contradicts the analytical prediction from Appendix D and the simulation results in the main text.

Appendix F Invasion of large-effect mutants into a two-strain resident

Let the population consists of two resident strains $Z = \langle Z_1, Z_2 \rangle$ with two-dimensional phenotypes, i.e., $Z_i = \langle z_1^{(i)}, z_2^{(i)} \rangle$. With quadratic cost and benefit functions, the population diversifies into a cooperator (with phenotype $z_{\max}$) and a defector (with phenotype 0) for each individual game. Results from individual-based simulations suggest that gradual evolution with small mutations in two dimensions (Fig 9) results in one of two possible resident populations: either a cooperator and a defector, $Z_{\mathrm{CD}} = \langle \langle 0, 0 \rangle, \langle z_{\max}, z_{\max} \rangle \rangle$, or two complementary specialists, $Z_{\mathrm{DOL}} = \langle \langle 0, z_{\max} \rangle, \langle z_{\max}, 0 \rangle \rangle$.

At the equilibrium frequencies $\hat{n} = \langle \hat{n}_1, \hat{n}_2 \rangle$, all strategies have the same expected payoff $\overline{P}$, i.e.,

$$\overline{P} J = \mathbf{P} \hat{n}, \tag{F.1}$$

where $P_{i,j}$ is the payoff of strain i upon interacting with strain j and J is a column vector of ones. Because $\overline{P}$ is a scalar value, we can re-write this equation as

$$\hat{n} = \overline{P} \mathbf{P}^{-1} J. \tag{F.2}$$

Since $\hat{n}$ is the vector of frequencies, the sum of all elements of $\hat{n}$ is one, i.e.,

$$J^T \hat{n} = \overline{P} J^T \mathbf{P}^{-1} J = 1. \tag{F.3}$$

Therefore,

$$\overline{P} = \frac{1}{J^T \mathbf{P}^{-1} J}. \tag{F.4}$$

Hence, the equilibrium frequency distribution is equal to

$$\hat{n} = \frac{\mathbf{P}^{-1} J}{J^T \mathbf{P}^{-1} J}, \tag{F.5}$$

which is proportional to $\mathbf{P}^{-1} J$.

We now investigate the success of large-effect mutants occupying the empty corners of the parameter space.

The CD state is stable against the invasion of either specialist mutant. For the resident configuration Z_{CD}, we have the payoff matrix

$$\mathbf{P}_{CD} = \begin{bmatrix} 0 & B(z_{max}) \\ B(z_{max}) - C(z_{max}) & B(2z_{max}) - C(z_{max}) \end{bmatrix}, \tag{F.6}$$

which results in equilibrium frequencies

$$\begin{aligned} \hat{n}_1 &= \frac{B(z_{max}) - B(2z_{max}) + C(z_{max})}{2B(z_{max}) - B(2z_{max})} \\ \hat{n}_2 &= \frac{B(z_{max}) - C(z_{max})}{2B(z_{max}) - B(2z_{max})}. \end{aligned} \tag{F.7}$$

The mean payoff of a mutant $Z' = \langle z'_1, z'_2 \rangle$ is given by

$$\begin{aligned} \overline{P}(Z', Z_{CD}) &= \hat{n}_1 \Big[(1-\alpha)\Big(B(z'_1) - C(z'_1)\Big) + \alpha\Big(B(z'_2) - C(z'_2)\Big)\Big] \\ &+ \hat{n}_2 \Big[(1-\alpha)\Big(B(z_{max} + z'_1) - C(z'_1)\Big) + \alpha\Big(B(z_{max} + z'_2) - C(z'_2)\Big)\Big]. \end{aligned} \tag{F.8}$$

Plugging in $\hat{n}_1$ and $\hat{n}_2$ from Eq F.7, $\alpha = 1/2$, $(z'_1 = 0, z'_2 = z_{max})$ or $(z'_1 = 0, z'_2 = z_{max})$, and noting that $B(0) = C(0) = 0$, we find

$$\overline{P}(Z', Z_{CD}) = \frac{B(z_{max})(B(z_{max}) - C(z_{max}))}{2B(z_{max}) - B(2z_{max})}. \tag{F.9}$$

Using a similar approach, we can calculate the mean payoff of a resident, say $\langle 0, 0 \rangle$. We take the right-hand side of Eq F.8 and replace 0 for z'_1 and for z'_2. Plugging in $\hat{n}_1$ and $\hat{n}_2$ from Eq F.7, $\alpha = 1/2$, and noting that $B(0) = C(0) = 0$, we find that the mean payoff of the resident exactly equals the right-hand side of Eq F.9. Since the resident and the mutant have identical mean payoffs, a rare mutant is not favored by natural selection and can only invade the resident population by neutral drift.

The DOL state is vulnerable to invasion by a cooperator or by a defector, depending on parameter choice. For the resident configuration Z_{DOL}, we have the payoff matrix

$$\mathbf{P}_{DOL} = \begin{bmatrix} \alpha B(2z_{max}) - \alpha C(z_{max}) & B(z_{max}) - \alpha C(z_{max}) \\ B(z_{max}) - (1-\alpha)C(z_{max}) & (1-\alpha)(B(2z_{max}) - C(z_{max})) \end{bmatrix}, \tag{F.10}$$

which, for $\alpha = 1/2$, results in equally frequent strains, $\hat{n}_1 = \hat{n}_2 = 1/2$ (which is a consequence of the model's symmetry).

Again assuming $\alpha = 1/2$, the mean payoff of a specialist (resident) strain is given by

$$\begin{aligned}\overline{P}(\langle 0, z_{\text{max}}\rangle, Z_{\text{DOL}}) &= \frac{1}{2}\left(\frac{B(z_{\text{max}}) - C(z_{\text{max}})}{2} + \frac{B(z_{\text{max}})}{2}\right) + \frac{1}{2}\left(\frac{B(2z_{\text{max}}) - C(z_{\text{max}})}{2}\right) \\ &= \frac{B(2z_{\text{max}}) + 2B(z_{\text{max}}) - 2C(z_{\text{max}})}{4}. \qquad \text{(F.11)}\end{aligned}$$

The mean payoff of a defector mutant is given by

$$\begin{aligned}\overline{P}(\langle 0, 0\rangle, Z_{\text{DOL}}) &= \frac{1}{2}\left(\frac{B(z_{\text{max}})}{2}\right) + \frac{1}{2}\left(\frac{B(z_{\text{max}})}{2}\right) \\ &= \frac{B(z_{\text{max}})}{2}, \qquad \text{(F.12)}\end{aligned}$$

and the mean payoff of a cooperator mutant is given by

$$\begin{aligned}\overline{P}(\langle z_{\text{max}}, z_{\text{max}}\rangle, Z_{\text{DOL}}) &= \frac{1}{2}\left(\frac{B(2z_{\text{max}}) - C(z_{\text{max}})}{2} + \frac{B(z_{\text{max}}) - C(z_{\text{max}})}{2}\right) + \frac{1}{2}\left(\frac{B(z_{\text{max}}) - C(z_{\text{max}})}{2} + \frac{B(2z_{\text{max}}) - C(z_{\text{max}})}{2}\right) \\ &= \frac{B(z_{\text{max}}) - B(2z_{\text{max}}) - 2C(z_{\text{max}})}{2}, \qquad \text{(F.13)}\end{aligned}$$

The defector mutant can invade whenever

$$\begin{aligned}\overline{P}(\langle 0, 0\rangle, Z_{\text{DOL}}) &> \overline{P}(\langle 0, z_{\text{max}}\rangle, Z_{\text{DOL}}) \\ 2C(z_{\text{max}}) &> B(2z_{\text{max}}) \\ 0 &< z_{\text{max}}(c - a + z_{\text{max}}(2ab - cd)). \qquad \text{(F.14)}\end{aligned}$$

Since z_{max} is always positive and the branching condition implies $cd < 2ab < 2cd$, it follows that the defector can invade whenever

$$\begin{aligned}z_{\text{max}} &> \frac{a - c}{2ab - cd} = 2z^{\star} \\ \frac{z_{\text{max}}}{2} &> z^{\star}. \qquad \text{(F.15)}\end{aligned}$$

The cooperator mutant can invade whenever $\overline{P}(\langle z_{\text{max}}, z_{\text{max}}\rangle, Z_{\text{DOL}}) > \overline{P}(\langle 0, z_{\text{max}}\rangle, Z_{\text{DOL}})$, which by an identical calculation is true whenever $z_{\text{max}} < z^{\star}$.

Both three-strain configurations are stable against invasion by a fourth strain. We can use the same procedure that we used above to show that the invasion fitness of a defector mutant in a resident population consisting of two specialists and a defector as well as the the invasion fitness of a cooperator mutant in a resident population consisting of two specialists and a cooperator are equal to zero. Hence, a fourth strain is not favored by natural selection and could only invade by genetic drift. See the *Mathematica* notebook `AppendixF.nb`, available in the GitHub repository,

for the full derivation (for readers without access to this proprietary software, we also included a `.pdf` version of the same file).

References

1. Ito HC, Dieckmann U. A New Mechanism for Recurrent Adaptive Radiations. Am Nat 2007;170:96–111.
2. Fehlberg E. Low-order classical Runge-Kutta formulas with stepsize control and their application to some heat transfer problems. Tech. rep. Washington, DC: NASA Technical Report 315, 1969.
3. Press WH, Teukolsky SA. Adaptive Stepsize Runge-Kutta Integration. Comput Phys 1992;6:191.
4. Ito HC, Dieckmann U. Evolutionary-branching lines and areas in bivariate trait spaces. Evol Ecol Res 2012;14:555–82.
5. Ito HC, Dieckmann U. Evolutionary branching under slow directional evolution. J Theor Biol 2014;360:290–314.
